# Alogliptin after acute coronary syndrome in patients with type 2 diabetes: a renal function stratified analysis of the EXAMINE trial

**DOI:** 10.1186/s12916-020-01616-8

**Published:** 2020-06-04

**Authors:** João Pedro Ferreira, Cyrus Mehta, Abhinav Sharma, Steven E. Nissen, Patrick Rossignol, Faiez Zannad

**Affiliations:** 1 Université de Lorraine, Centre d’Investigations Cliniques Plurithématique Inserm 1433, Nancy, France, CHRU de Nancy, Inserm U1116, Nancy, France, FCRIN INI-CRCT, Nancy, France; 2 INSERM U1116, CHRU, F-CRIN INI-CRCT (Cardiovascular and Renal Clinical Trialists), 4 rue du Morvan, 54500 Vandoeuvre les Nancy, Nancy, France; 3 Cytel Corportation, Cambridge, MA USA; 4 Harvard T.H. Chan School of Public Health, Boston, MA USA; 5 Division of Cardiology, McGill University Health Centre, McGill University, Montreal, Quebec Canada; 6 Cleveland Clinic Coordinating Center for Clinical Research, Department of Cardiovascular Medicine, Cleveland Clinic, Cleveland, OH USA

**Keywords:** Alogliptin, Renal function, Stratification, Outcomes

## Abstract

**Background:**

The EXAMINE trial tested the efficacy and safety of alogliptin, an inhibitor of dipeptidyl peptidase 4, compared with placebo in 5380 patients with type 2 diabetes and a recent acute coronary syndrome. Because alogliptin is cleared by the kidney, patients were stratified according to screening renal function within two independently randomized strata: (1) estimated glomerular filtration rate (eGFR) ≥ 60 ml/min/1.73m^2^ and (2) eGFR < 60 ml/min/1.73m^2^. We aim to assess the efficacy and safety of alogliptin vs. placebo according to the renal function strata.

**Methods:**

Cox-proportional hazard models with an interaction term by renal function strata were used. The primary endpoint was a composite of cardiovascular death, nonfatal myocardial infarction (MI), or nonfatal stroke.

**Results:**

Patient characteristics were balanced within each renal function strata. In total, 3946 patients were randomized within the eGFR ≥ 60 stratum, and 1434 patients within the eGFR < 60 stratum. The effect of alogliptin was modified by the renal function strata. Primary outcome: eGFR ≥ 60 HR = 0.81, 95%CI, 0.65–0.99, and eGFR < 60 HR = 1.20, 95%CI, 0.95–1.53; interaction_p_ = 0.014. Cardiovascular death: eGFR ≥ 60 HR = 0.61, 95%CI, 0.42–0.88, and eGFR < 60 HR = 1.16, 95%CI, 0.82–1.65; interaction_p_ = 0.013. Non-fatal MI: eGFR ≥ 60 HR = 0.86, 95%CI, 0.66–1.13, and eGFR < 60 HR = 1.48, 95%CI, 1.07–2.06; interaction_p_ = 0.013.

**Conclusions:**

Alogliptin may benefit patients with eGFR ≥ 60, but may be detrimental to patients with eGFR < 60 ml/min/1.73m^2^. These hypothesis-generating findings require further validation to assess the potential benefit and risk of alogliptin across the renal function spectrum among patients with type 2 diabetes and a recent acute coronary syndrome.

**Trial registration:**

ClinicalTrials.gov, NCT00968708

## Background

Type 2 diabetes epidemic is a global public health issue and is strongly associated with atherosclerotic cardiovascular disease [1]. To date, few drugs have demonstrated their efficacy and safety according to baseline renal function, which may impact treatment decisions in a personalized manner [2]. Concerns regarding adverse cardiovascular outcomes with antidiabetic agents [3, 4] prompted the Food and Drug Administration (FDA) to issue guidance (in December 2008) that included specific requirements for cardiovascular safety assessment before and after the approval of new antidiabetic therapies [5]. Regulatory agencies in other countries have adopted similar policies.

Alogliptin is a selective inhibitor of dipeptidyl peptidase 4 (DPP-4) approved for the treatment of type 2 diabetes [6]. The Examination of Cardiovascular Outcomes with Alogliptin versus Standard of Care (EXAMINE) trial sought to determine whether alogliptin was noninferior to placebo with respect to major cardiovascular events in patients with type 2 diabetes with recent acute coronary syndrome (a very high cardiovascular risk population) [7]. In the overall population, alogliptin significantly reduced glycated hemoglobin without increasing the rates of major adverse cardiovascular events [7, 8].

Because renal function is strongly associated with outcomes in diabetic populations and alogliptin is predominantly cleared by the kidney [9–11], the EXAMINE trial had a prespecified stratification according to the estimated glomerular filtration rate (eGFR) at screening. The two independently randomized strata were (1) eGFR ≥ 60 ml/min/1.73m^2^ and (2) eGFR < 60 ml/min/1.73m^2^, reflecting patients with normal/near normal and impaired renal function, respectively [12].

The aim of the present analysis is to assess the effect of alogliptin according to the screening renal function strata.

## Methods

### Study design

Details of the EXAMINE study design were previously published [7, 12]. In short, the EXAMINE trial was a multicenter, randomized, double-blind trial. The steering committee, consisting of academic members and three nonvoting representatives of the sponsor (Takeda Development Center Americas), designed and oversaw the conduct of the trial. An independent data and safety monitoring committee monitored the trial and had access to the unblinded data. The appropriate national and institutional regulatory authorities and ethics committees approved the study design, and all participants provided written informed consent.

### Study patients

Patients were eligible for enrolment if they had received a diagnosis of type 2 diabetes mellitus, were receiving antidiabetic therapy (other than a DPP-4 inhibitor or GLP-1 analogue), and had had an acute coronary syndrome within 15 to 90 days before randomization. Further criteria for the diagnosis of type 2 diabetes included a glycated hemoglobin level of 6.5 to 11.0% at screening, or if the antidiabetic regimen included insulin, a glycated hemoglobin level of 7.0 to 11.0%. Acute coronary syndromes included acute myocardial infarction and unstable angina requiring hospitalization. Major exclusion criteria were a diagnosis of type 1 diabetes, unstable cardiac disorders (e.g., New York Heart Association class IV heart failure, refractory angina, uncontrolled arrhythmias, critical valvular heart disease, or severe uncontrolled hypertension), and dialysis within 14 days before screening.

### Study treatment

Patients were randomly assigned to receive alogliptin or placebo, administered in a double-blind fashion, in addition to standard-of-care treatment for type 2 diabetes mellitus. Throughout the study, patients were required to receive standard-of-care treatment for type 2 diabetes and cardiovascular risk factors according to regional guidelines.

### Stratification according to renal function

Stratification according to eGFR (calculated with the use of the Modification of Diet in Renal Disease formula [13]) was performed at screening which that occurred 9 (7–13) days before the randomization. In the screening visit, patients were allocated to one of the following strata: (1) eGFR ≥ 60 ml/min/1.73m^2^ (eGFR ≥ 60) and (2) eGFR < 60 ml/min/1.73m^2^ (eGFR < 60). As prespecified, patients were randomized within each stratum.

Because alogliptin is cleared by the kidney, the doses of alogliptin (and matching placebo) were modified according to kidney function at the time of randomization and could be adjusted during the post-randomization period. The daily doses of the study drug were as follows: 25 mg in patients with an eGFR ≥ 60 ml/min/1.73m^2^, 12.5 mg in patients with an eGFR of 30 to < 60 ml/min/1.73m^2^, and 6.25 mg in patients with an eGFR < 30 ml/min/1.73m^2^.

### Endpoints

The primary endpoint was a composite of death from cardiovascular causes, nonfatal myocardial infarction (MI), or nonfatal stroke (3-point major adverse cardiovascular events [MACE]). The principal secondary endpoint was the primary composite endpoint with the addition of urgent revascularization due to unstable angina within 24 h after hospital admission. Exploratory endpoints included death from cardiovascular causes and death from any cause. The endpoint of hospital admission for heart failure (HF) was defined as an inpatient admission or an emergency department visit of more than 12 h with clinical manifestations of HF, including new or worsening dyspnea, orthopnea, paroxysmal nocturnal dyspnea, peripheral edema, bibasilar rales on pulmonary examination, jugular venous distention, new third heart sound, radiographic evidence of HF, and parenteral diuretic, inotropic, or vasodilator therapy, ultrafiltration or dialysis, or mechanical or surgical intervention (including heart transplant). Safety endpoints included angioedema, hypoglycemia, pancreatitis, cancer, and the results of laboratory testing. An independent central adjudication committee adjudicated all suspected primary endpoint events and other cardiovascular endpoints, as well as all deaths.

### Statistical analysis

All analyses were done by the intention to treat principle. Baseline characteristics were summarized as frequencies for categorical variables and mean ± standard deviation (SD) or median (percentile 25–75) according to the variables’ distribution on the histogram. Time to the first occurrence of an endpoint component was analyzed with Cox proportional hazards model within each screening renal function stratum and in the whole population with an interaction term on the eGFR strata for assessing subgroup heterogeneity. Cumulative event rates per 100-person years are also reported. For studying the treatment effect on myocardial infarction, a competing risk model was used, using death as competing event as described by Fine and Gray [14]. Changes from baseline to the last visit in glycated hemoglobin (HbA1c), HDL, LDL, total cholesterol, triglycerides, eGFR, and urinary albumin-to-creatinine ration (UACR) were assessed with an ANCOVA model with terms for treatment, renal function at baseline, and the corresponding baseline HbA1c values as covariates and reported within each eGFR stratum. Repeated measures were available for HBA1c, LDL, HDL, total cholesterol, triglycerides, body mass index, creatinine, eGFR, UACR, systolic and diastolic blood pressure, and C-reactive protein. The between treatment arm differences in the changes of these parameters along time (months) were assessed (within each screening renal function stratum) using repeated measures multilevel mixed-effects linear regression models, with time, treatment, and an interaction of treatment by time as fixed effects and random-effects at the patient level. All statistical analyses were assessed at a two-sided significance level of 5%, and all confidence intervals (CIs) were reported as two-sided values with a confidence level of 95%. No adjustments were made to the nominal *p* values for multiple testing. All statistical analyses were performed with Stata (StataCorp®, version 16.1). This study is registered with ClinicalTrials.gov, number NCT00968708.

## Results

### Study patients by renal function strata

A total of 5380 patients were recruited: 3946 within the eGFR ≥ 60 stratum and 1434 within the eGFR < 60 stratum. As per-strata procedure, the groups were well balanced with regard to their characteristics and nonstudy medications within each stratum (Table 1). The comparison of patients’ characteristics between the eGFR strata are presented in Additional file: Table S1. Compared with patients in the eGFR ≥ 60 stratum, those in the eGFR < 60 stratum were older, had longer duration of diabetes, and had more comorbid conditions. The median duration of follow-up was 533 days (pct_25–75_ 280–751).

**Table 1:** Baseline characteristics of the study population by screening renal function strata and study drug

Renal function strata	eGFR ≥ 60 stratum			eGFR < 60 stratum		
Study drug	Placebo	Alogliptin	*p* value	Placebo	Alogliptin	*p* value
*N*	1963	1983		716	718	
Age (years), mean ± SD	58.9 ± 9.3	59.1 ± 9.5	0.40	65.8 ± 9.7	66.2 ± 9.4	0.43
Age < 65 years	1424 (72.5%)	1419 (71.6%)	0.49	321 (44.8%)	309 (43.0%)	0.49
Age ≥ 65 years	539 (27.5%)	564 (28.4%)		395 (55.2%)	409 (57.0%)	
Male sex	1427 (72.7%)	1413 (71.3%)	0.31	396 (55.3%)	415 (57.8%)	0.34
Diabetes duration (years), median (IQR)	6.4 (2.5, 12.0)	6.2 (2.2, 11.7)	0.22	10.0 (4.3, 16.8)	10.4 (4.4, 17.3)	0.50
BMI (kg/m^2^), mean ± SD	29.6 ± 5.7	29.5 ± 5.3	0.59	29.3 ± 5.9	29.2 ± 5.7	0.56
Race			0.55			0.98
White	1460 (74.4%)	1475 (74.4%)		483 (67.5%)	491 (68.4%)	
Asian	360 (18.3%)	369 (18.6%)		182 (25.4%)	178 (24.8%)	
Black	88 (4.5%)	74 (3.7%)		27 (3.8%)	27 (3.8%)	
Other	55 (2.8%)	65 (3.3%)		24 (3.4%)	22 (3.1%)	
Geographic region			1.00			1.00
United States, Canada	312 (15.9%)	314 (15.8%)		114 (15.9%)	113 (15.7%)	
Mexico, Central/South America	502 (25.6%)	512 (25.8%)		191 (26.7%)	188 (26.2%)	
Western Europe, Australia, New Zealand, Middle East	234 (11.9%)	240 (12.1%)		69 (9.6%)	73 (10.2%)	
Eastern Europe, Africa	582 (29.6%)	580 (29.2%)		171 (23.9%)	175 (24.4%)	
Asia/Pacific	333 (17.0%)	337 (17.0%)		171 (23.9%)	169 (23.5%)	
Smoking	327 (16.7%)	300 (15.1%)	0.19	56 (7.8%)	51 (7.1%)	0.60
Hypertension	1586 (80.8%)	1590 (80.2%)	0.63	654 (91.3%)	639 (89.0%)	0.14
Previous MI	1711 (87.2%)	1748 (88.1%)	0.35	634 (88.5%)	641 (89.3%)	0.66
PCI	1253 (63.8%)	1248 (62.9%)	0.56	430 (60.1%)	441 (61.4%)	0.60
CABG	221 (11.3%)	237 (12.0%)	0.50	120 (16.8%)	110 (15.3%)	0.46
HF history	480 (24.5%)	495 (25.0%)	0.71	282 (39.4%)	276 (38.4%)	0.71
Previous stroke	119 (6.1%)	115 (5.8%)	0.73	74 (10.3%)	80 (11.1%)	0.62
PAD	143 (7.3%)	166 (8.4%)	0.20	109 (15.2%)	96 (13.4%)	0.31
AFib	108 (5.5%)	101 (5.1%)	0.57	79 (11.0%)	88 (12.3%)	0.47
eGFR* (ml/min/1.73m^2^), mean ± SD	79.7 ± 16.9	79.3 ± 16.8	0.43	47.2 ± 13.7	47.6 ± 13.8	0.57
eGFR* < 60 ml/min/1.73m^2^	194 (9.9%)	171 (8.6%)	0.17	599 (83.7%)	601 (83.7%)	0.98
eGFR < 30 ml/min/1.73m^2^	–	–	–	77 (10.8%)	77 (10.8%)	0.99
Index ACS Event Type			0.76			0.57
Myocardial Infarction	1513 (77.3%)	1521 (76.9%)		555 (77.6%)	563 (78.9%)	
Unstable Angina	445 (22.7%)	458 (23.1%)		160 (22.4%)	151 (21.1%)	
Time from index ACS to randomization, median (IQR)	45.0 (29.0, 63.0)	44.0 (30.0, 63.0)	0.68	44.0 (30.0, 65.0)	43.0 (29.0, 66.0)	0.56
Troponin I (ng/L), median (IQR)	7.8 (4.3, 16.5)	7.7 (3.8, 17.0)	0.44	12.8 (6.4, 27.7)	14.0 (7.1, 30.2)	0.08
Heart rate (bpm), mean ± SD	71.4 ± 10.8	71.6 ± 10.4	0.56	70.7 ± 11.1	71.0 ± 11.4	0.55
SBP (mmHg), mean ± SD	128.5 ± 16.7	127.9 ± 15.8	0.21	131.1 ± 17.6	131.2 ± 17.2	0.93
DBP (mmHg), mean ± SD	76.9 ± 9.5	76.6 ± 9.4	0.41	75.5 ± 10.1	75.4 ± 10.3	0.95
Total cholesterol (mg/dL), mean ± SD	153.5 ± 42.2	151.7 ± 43.1	0.19	158.6 ± 46.4	159.8 ± 46.6	0.62
LDL cholesterol (mg/dL), mean ± SD	78.1 ± 33.6	76.6 ± 33.2	0.17	81.0 ± 37.2	83.4 ± 38.6	0.24
HDL cholesterol (mg/dL), mean ± SD	42.8 ± 10.0	43.2 ± 10.7	0.25	43.8 ± 11.1	43.1 ± 11.2	0.25
Triglycerides (mg/dL), mean ± SD	165.5 ± 108.7	160.9 ± 105.5	0.18	168.8 ± 98.2	167.7 ± 90.8	0.84
UACR (mg/g creat.), mean ± SD	17.6 ± 54.1	18.1 ± 69.1	0.83	67.7 ± 152.1	74.1 ± 166.6	0.53
C-reactive protein (mg/dL), mean ± SD	5.1 ± 12.1	5.1 ± 12.5	0.94	7.0 ± 14.9	6.8 ± 18.8	0.79
Antiplatelet agents	1911 (97.4%)	1933 (97.5%)	0.80	691 (96.5%)	697 (97.1%)	0.54
Beta-blockers	1624 (82.7%)	1623 (81.8%)	0.47	579 (80.9%)	585 (81.5%)	0.77
ACEi/ARBs	1641 (83.6%)	1626 (82.0%)	0.18	569 (79.5%)	575 (80.1%)	0.77
Statins	1796 (91.5%)	1798 (90.7%)	0.37	624 (87.2%)	648 (90.3%)	0.064
Antidiabetic agents	1948 (99.2%)	1968 (99.2%)	0.98	701 (97.9%)	708 (98.6%)	0.31
Insulin	550 (28.0%)	526 (26.5%)	0.29	262 (36.6%)	267 (37.2%)	0.82
Metformin	1437 (73.2%)	1424 (71.8%)	0.33	368 (51.4%)	333 (46.4%)	0.057
Thiazolidinediones	42 (2.1%)	41 (2.1%)	0.87	22 (3.1%)	26 (3.6%)	0.56
Sulfonylureas	927 (47.2%)	946 (47.7%)	0.76	310 (43.3%)	320 (44.6%)	0.63
Calcium channel blockers	388 (19.8%)	375 (18.9%)	0.50	223 (31.1%)	211 (29.4%)	0.47
Diuretics (any)	616 (31.4%)	624 (31.5%)	0.95	393 (54.9%)	381 (53.1%)	0.49

Stratification according to renal function was performed at the baseline visit, as follows: (1) “normal renal function” stratum if eGFR ≥60 ml/min/1.73m^2^ or (2) “impaired renal function” stratum if eGFR < 60 ml/min/1.73m^2^

*MI* myocardial infarction, *PCI* percutaneous coronary intervention, *CABG* coronary-artery bypass grafting, *HF* heart failure, *PAD* peripheral artery disease, *AFib* atrial fibrillation, *eGFR* estimated glomerular filtration rate, *ACS* acute coronary syndrome, *SBP* systolic blood pressure, *DBP* diastolic blood pressure, *ACEi/ARBs* angiotensin converting enzyme inhibitors/angiotensin receptor blockers, *UACR* urinary albumin-to-creatinine ratio

*eGFR at randomization, that occurred 9 (7–13) days after the baseline visit

**Fig. 1:**
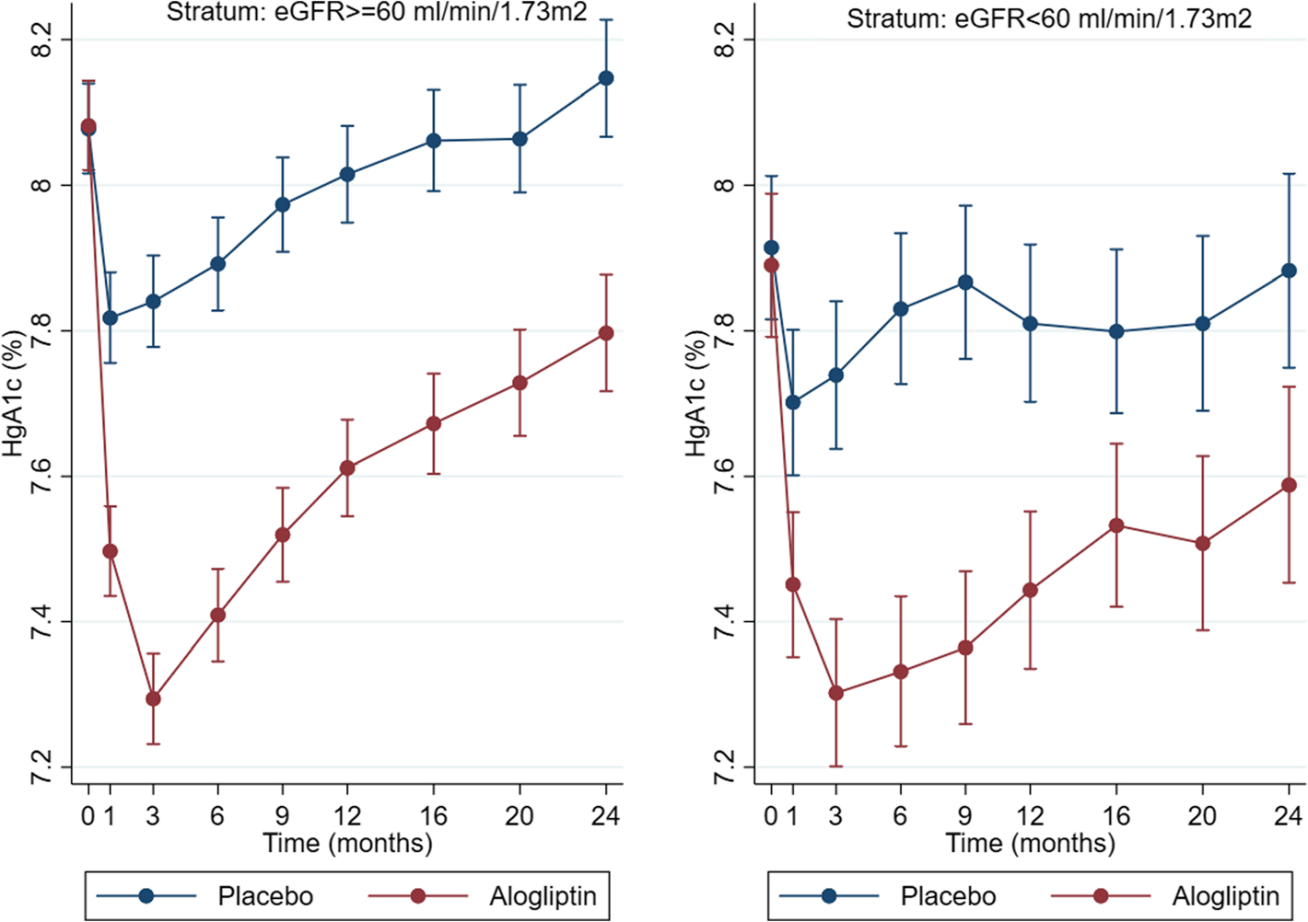
Glycated hemoglobin levels over time by study treatment and renal function strata. *p* value < 0.001 for all time point comparisons within each eGFR stratum. Global *p* value for between eGFR strata interaction = 0.61. HgA1c, glycated hemoglobin; eGFR, estimated glomerular filtration rate

Changes in HbA1c levels over time are shown in Fig. 1. The mean HbA1c change from baseline was similar in both renal function strata: − 0.27% in the alogliptin group vs. + 0.05% in the placebo group within the eGFR ≥ 60 stratum and − 0.30% in the alogliptin group vs. + 0.05% in the placebo group within the eGFR < 60 stratum; *p* for between eGFR strata interaction = 0.61. The between treatment arm HbA1c differences were significant at each studied time point (*p* < 0.001 for all time point comparisons). Within strata changes (from baseline to the last visit) in HbA1c, HDL, LDL, total cholesterol, triglycerides, eGFR, and UACR did not present major differences between alogliptin and placebo groups (Additional file: Table S2). Within each eGFR stratum, the between treatment arm differences in the repeated measures of total, LDL, and HDL cholesterol; body mass index; creatinine; eGFR; UACR; systolic and diastolic blood pressure; and C-reactive protein were not significantly different after adjusting for multiple comparisons at each studied time point (*p* > 0.05 for all comparisons; Additional file: Figure S1).

### Primary and secondary endpoints by renal function strata

**Fig. 2:**
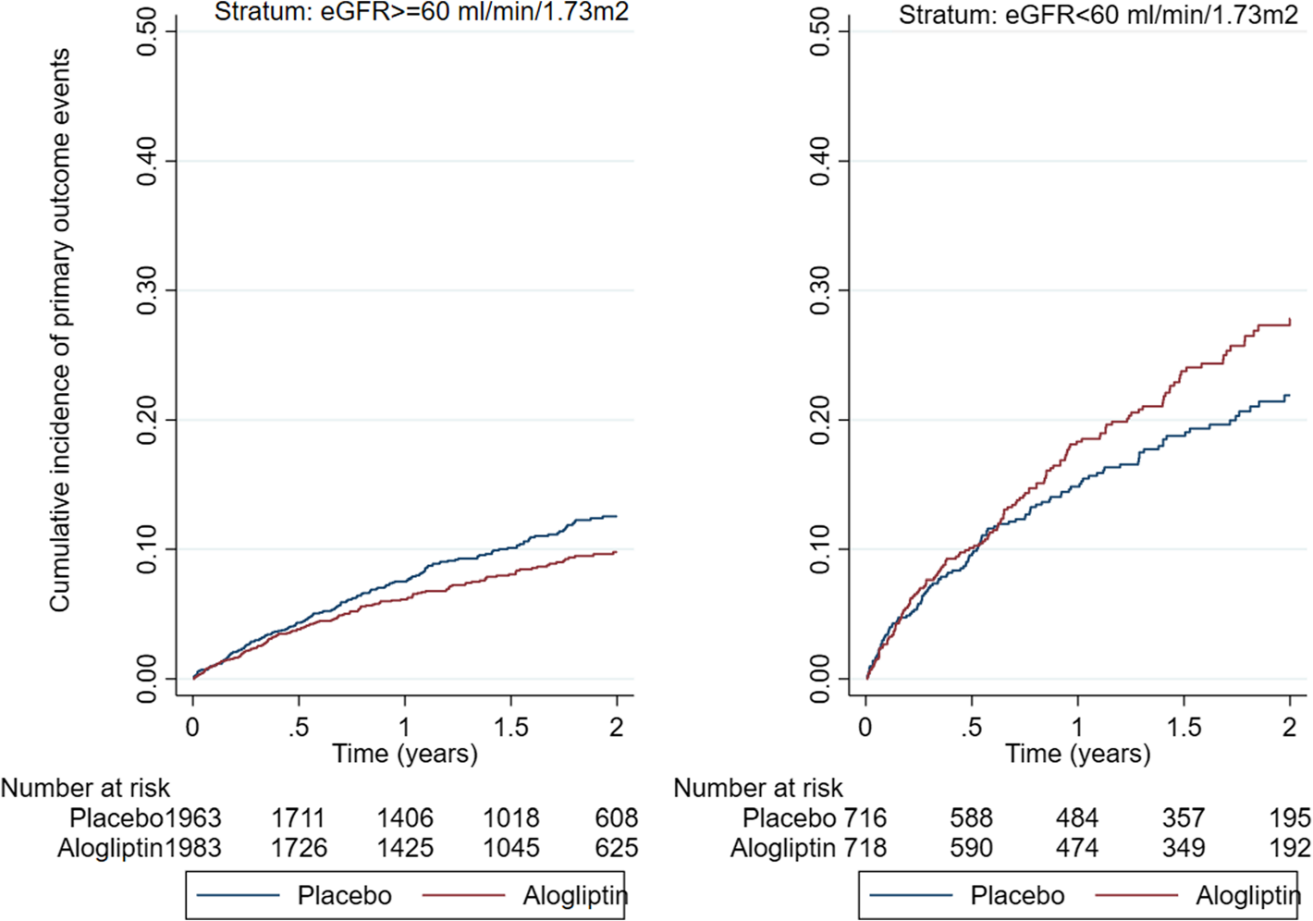
Cumulative incidence Kaplan-Meier curves by study treatment and renal function strata. *p* value for between eGFR strata interaction = 0.014. eGFR, estimated glomerular filtration rate

Within the eGFR ≥ 60 stratum, the primary outcome occurred in 192 (9.8%) patients in the placebo group and 157 (7.9%) patients in the alogliptin group, HR (95%CI) = 0.81 (0.65–0.99), whereas within the eGFR < 60 stratum, the primary outcome occurred in 124 (17.3%) patients in the placebo group and 148 (20.6%) patients in the alogliptin group, HR (95%CI) = 1.20 (0.95–1.53); *p* for interaction between renal function strata = 0.014 (Fig. 2). Within the eGFR ≥ 60 stratum, the principal secondary outcome occurred in 226 (11.5%) patients in the placebo group and 189 (9.5%) patients in the alogliptin group, HR (95%CI) = 0.82 (0.68–0.99), whereas within the eGFR< 60 stratum, the principal secondary outcome occurred in 133 (18.6%) patients in the placebo group and 155 (21.6%) patients in the alogliptin group, HR (95%CI) = 1.17 (0.93–1.47); *p* for interaction between renal function strata = 0.021 (Table 2 (A)). The components of the primary endpoint and the totality of the fatal events are presented in Table 2 (B). Within the eGFR ≥ 60 stratum, non-fatal myocardial infarction occurred in 113 (5.8%) patients in the placebo group and 99 (5.0%) patients in the alogliptin group, HR (95%CI) = 0.86 (0.66–1.13), whereas within eGFR < 60 stratum, non-fatal myocardial infarction occurred in 60 (8.4%) patients in the placebo group and 88 (12.3%) patients in the alogliptin group, HR (95%CI) = 1.48 (1.07–2.06); *p* for interaction = 0.013. Within the eGFR ≥ 60 stratum, cardiovascular death occurred in 72 (3.7%) patients in the placebo group and 44 (2.2%) patients in the alogliptin group, HR (95%CI) = 0.61 (0.42–0.88), whereas within the eGFR < 60 stratum, cardiovascular death occurred in 58 (8.1%) patients in the placebo group and 68 (9.5%) patients in the alogliptin group, HR (95%CI) = 1.16 (0.82–1.65); *p* for interaction between renal function strata = 0.013. No treatment-by-eGFR strata interactions were present for all-cause death, heart failure hospitalizations, and non-fatal stroke **(**Table 2 (B)). These results were confirmed using models that account for competing events. The respective event rates (per 100 person-years) and absolute rate differences are depicted in Additional file: Table S3.

**Table 2:** Study endpoints by renal function strata

(A) Primary and secondary endpoints by renal function strata					
**Endpoint**	**Renal function**	**Placebo**	**Alogliptin**	**HR (95% CI)**	**Interaction*p*value**
Primary	eGFR ≥ 60	192 (9.8%)	157 (7.9%)	0.81 (0.65–0.99)	0.014
	eGFR < 60	124 (17.3%)	148 (20.6%)	1.20 (0.95–1.53)	
Secondary	eGFR ≥ 60	226 (11.5%)	189 (9.5%)	0.82 (0.68–0.99)	0.021
	eGFR < 60	133 (18.6%)	155 (21.6%)	1.17 (0.93–1.47)	
(B) Components of primary endpoint and other endpoints by renal function strata					
**Components of primary endpoint**	**Renal function**	**Placebo**	**Alogliptin**	**HR (95% CI)**	**Interaction*p*value**
CV death	eGFR ≥ 60	60 (3.1%)	37 (1.9%)	0.61 (0.41–0.92)	0.079
	eGFR < 60	51 (7.1%)	52 (7.2%)	1.01 (0.69–1.48)	
Non-fatal MI*	eGFR ≥ 60	113 (5.8%)	99 (5%)	0.86 (0.66–1.13)	0.013
	eGFR < 60	60 (8.4%)	88 (12.3%)	1.48 (1.07–2.06)	
Non-fatal stroke	eGFR ≥ 60	19 (1%)	21 (1.1%)	1.20 (0.95–1.53)	0.28
	eGFR < 60	13 (1.8%)	8 (1.1%)	0.61 (0.25–1.47)	
**Other endpoints**	**Renal function**	**Placebo**	**Alogliptin**	**HR (95% CI)**	**Interaction*p*value**
All-cause death	eGFR ≥ 60	89 (4.5%)	74 (3.7%)	0.82 (0.60–1.12)	0.56
	eGFR < 60	84 (11.7%)	79 (11.0%)	0.93 (0.68–1.27)	
All CV deaths	eGFR ≥ 60	72 (3.7%)	44 (2.2%)	0.61 (0.42–0.88)	0.013
	eGFR < 60	58 (8.1%)	68 (9.5%)	1.16 (0.82–1.65)	
HF hospitalizations	eGFR ≥ 60	43 (2.2%)	44 (2.2%)	1.01 (0.67–1.55)	0.32
	eGFR < 60	46 (6.4%)	62 (8.6%)	1.35 (0.92–1.97)	

The primary endpoint was a composite of cardiovascular death, non-fatal MI, and non-fatal stroke. The secondary endpoint was a composite of death from cardiovascular causes, nonfatal myocardial infarction, nonfatal stroke, or urgent revascularization due to unstable angina within 24 h after hospital admission

*MI* myocardial infarction, *CV* cardiovascular, *HF* heart failure, *eGFR* estimated glomerular filtration rate in ml/min/1.73 m2

*After adjustment for the competing risk of death, HR (95% CI) is 0.87 (0.66–1.14) for normal and 1.50 (1.08–2.08) for impaired renal function

### Primary and secondary endpoints by eGFR subgroups

The eGFR used for the prespecified stratification was performed at the screening visit that occurred 9 days (pct_25–75_ 7–13) before the randomization visit; 16% of patients stratified in the eGFR < 60 stratum improved their eGFR to ≥ 60 from the screening to the randomization visit, whereas 9% decreased their eGFR from ≥ 60 to < 60 ml/min/1.73m^2^ (Additional file: Table S4). In the main EXAMINE trial report [7], the eGFR used for the “subgroup-interaction” assessment was the eGFR from the randomization visit (and not the prespecified renal function stratification). Nevertheless, in the main report (*supplemental material*), a significant treatment by eGFR subgroup interaction was present: eGFR ≥ 60 HR = 0.84 (0–68-104) vs. eGFR < 60 HR = 1.15 (0.91–1.46); *p* for interaction = 0.046.

### Adverse events by renal function strata

Patients in the alogliptin group had more hypoglycemic events (but with low absolute proportions) in the eGFR≥60 stratum 6.1% vs. 4.6%, but not in the eGFR < 60 stratum: 8.5% vs. 11.5%; *p* for interaction = 0.007. Serious hypoglycemia was more frequently observed in patients with eGFR < 60, but the absolute rates were low (< 2%) and not different between the alogliptin and placebo groups; *p* for interaction = 0.14. Elevation of serum aminotransferases (> 3× the upper limit of normal) and other adverse events by treatment allocation did not differ between the renal function strata (Table 3).

**Table 3:** Safety endpoints

Adverse events	Placebo	Alogliptin	*p* value	Inter. *p*
***eGFR ≥ 60 stratum***				
Any serious adverse event	607 (30.9%)	564 (28.4%)	0.088	0.42
Serious hypoglycemia	4 (0.2%)	9 (0.5%)	0.17	0.14
Any adverse event	1490 (75.9%)	1532 (77.3%)	0.32	0.95
Any hypoglycemia	91 (4.6%)	120 (6.1%)	0.048	0.007
Pancreatitis				0.36
Acute	5 (0.3%)	5 (0.3%)	0.99	
Chronic	4 (0.2%)	2 (0.1%)	0.41	
Angioedema	6 (0.3%)	7 (0.4%)	0.80	0.78
Malignancy	30 (1.5%)	38 (1.9%)	0.35	0.28
Dialysis	3 (0.2%)	1 (0.1%)	0.31	0.27
Serum aminotransferases > 3× upper limit of normal at any time during trial				0.11
Alanine aminotransferase	37 (1.9%)	43 (2.2%)	0.53	
Aspartate aminotransferase	32 (1.6%)	27 (1.4%)	0.49	
***eGFR < 60 stratum***				
Any serious adverse event	345 (48.2%)	343 (47.8%)	0.88	0.42
Serious hypoglycemia	12 (1.7%)	9 (1.3%)	0.51	0.14
Any adverse event	621 (86.7%)	628 (87.5%)	0.68	0.95
Any hypoglycemia	82 (11.5%)	61 (8.5%)	0.062	0.007
Pancreatitis				0.36
Acute	3 (0.4%)	7 (1.0%)	0.21	
Chronic	0	3 (0.4%)	0.083	
Angioedema	7 (1.0%)	10 (1.4%)	0.45	0.78
Malignancy	21 (2.9%)	17 (2.4%)	0.51	0.28
Dialysis	19 (2.7%)	23 (3.2%)	0.54	0.27
Serum aminotransferases > 3× upper limit of normal at any time during trial				0.11
Alanine aminotransferase	9 (1.3%)	21 (2.9%)	0.027	
Aspartate aminotransferase	11 (1.5%)	21 (2.9%)	0.075	

*p* values were calculated by Fisher’s exact test with no adjustment for multiple comparisons. Hypoglycemia was reported by site investigators. The upper limit of normal for the alanine aminotransferase was25 U/L and for aspartate aminotransferase was 22 U/L

*eGFR* estimated glomerular filtration rate in ml/min/1.73 m^2^, *Inter. p p* value for interaction between eGFR strata and treatment allocation for each outcome in a logistic regression model

## Discussion

In this post hoc analysis of the EXAMINE trial, we evaluated the impact of randomization to alogliptin versus placebo within prespecified strata of renal function at the screening visit: eGFR ≥ 60 and eGFR < 60 ml/min/1.73m^2^. We found that randomization to alogliptin was associated with a reduction in the primary 3-point MACE outcome in participants within the eGFR ≥ 60 stratum but not among participants within the eGFR < 60 stratum who might have experienced an excess of adverse events, particularly MI.

Stratified randomization is recommended when there is a strong a priori expectation that the stratification variable is prognostic for treatment response. Treatment balance is thereby maintained within each stratum resulting in a more efficient comparison of treatment effect [15]. The inclusion of renal function as a stratification variable thus ensured a better balance between the treatment and placebo arms for both the eGFR ≥ 60 and eGFR < 60 strata. Moreover, because the stratification by renal function was identified at screening, before any data were observed, it lends greater weight to the results than the subsequent subgroup analysis.

Renal function is a major outcome driver and portends a high risk of future cardiovascular events in patients with and without diabetes [9, 10]. In the subgroup analyses reported within the primary results of the EXAMINE trial, the impact of alogliptin versus placebo on the primary outcome was presented based on the randomization (and not the screening) renal function [7]. Notwithstanding, a statistically significant treatment interaction by randomization eGFR subgroup was also observed [7]. The results between the two analyses (i.e., screening and randomization) are therefore directionally consistent.

Alogliptin could reduce the risk of cardiovascular death among patients within the eGFR ≥ 60 stratum but not in patients within the eGFR < 60 stratum, where an excess of adverse events, particularly MI, could be observed. Alogliptin is primarily eliminated by the kidney, with approximately 60–80% of the dose excreted unchanged in the urine [11]. In patients with renal impairment, alogliptin can have up to 3-fold higher accumulation [16]. The long-term, large-scale effects of alogliptin in patients with renal impairment remain largely unknown [16–18]. An analysis of the FDA Adverse Event Reporting System (FAERS) from 2006 to 2015 studying 13.4 million adverse event reports for diabetics and cardiovascular drugs found an excess for signals of disproportionate reporting of MI with alogliptin (compared with other “gliptins”) [15]. These large-scale registry data align with our randomized placebo-controlled data showing a 48% excess risk of myocardial infarction (on a relative scale) and + 2.8 events per 100 person-years (on an absolute scale) in patients allocated to alogliptin within the eGFR < 60 stratum. Data on the subgroup of patients with renal dysfunction are not shown in this FAERS report [15]; therefore, one cannot ascertain if the excess risk was driven by patients with renal impairment. To date, no other cardiovascular safety trial of a DPP-4 inhibitor versus placebo (or active comparator) has demonstrated an excess of risk of MACE with the DPP-4 inhibitor based on renal function [19–22].

The analyses of the reported adverse events and repeated measures of cholesterol, blood pressure, and renal function in the EXAMINE trial do not provide additional insight on the potential mechanisms underlying the increased rate of myocardial infarction with alogliptin within the eGFR < 60 stratum. Patients with eGFR < 60 had higher proportion of hepatic cytolysis with alogliptin (+ 1.6% serum alanine aminotransferase > 3× the upper limit of normal), but the absolute number of events was low (< 3%) and does not, per se, explain the observed risk. Serious hypoglycemia was also more frequently observed in patients with eGFR < 60, but the absolute rates were also low (< 2%) and not different between alogliptin and placebo. In concordance with other “gliptin” reports, alogliptin did not have major effect in albuminuria and renal function regardless of the renal function stratum. Natriuretic peptides were also not affected by alogliptin treatment [8, 23].

### Limitations

In the advent of a qualitative stratum by treatment interaction (as observed herein), then the estimate of the common hazard ratio obtained from the stratified Cox model cannot be interpreted and the better option is to report the hazard ratio estimates and hypothesis test results separately for the two strata (as here performed). It is of worth noting that in this context, the interaction tests for treatment by renal function were statistically significant for both the primary and secondary endpoints even though it is well known that such tests have low power [15]. Nevertheless, it is not possible to control the family-wise error rate of a joint hypothesis that involves both the treatment effect and the strata by treatment interaction since it was not prespecified in the EXAMINE trial statistical analysis plan as part of the hierarchical testing strategy, therefore these findings cannot be corrected for multiplicity of testing. Furthermore, the EXAMINE trial is underpowered to assess the treatment effect in each stratum separately and even less powered to assess the effects in patients with severely impaired renal function. For these reasons, the findings presented here should be regarded as “hypothesis generating” and further investigation is required, particularly to assess the safety of alogliptin in patients with renal dysfunction.

## Conclusion

Alogliptin may benefit patients with normal/near normal renal function, but may be detrimental to patients with impaired renal function. These hypothesis-generating findings require further validation to assess the potential benefit and risk of alogliptin across the renal function spectrum among patients with type 2 diabetes who had had a recent acute coronary syndrome.

## Supplementary information

**Additional file 1: Table S1.** Baseline characteristics of the study population by screening renal function strata. **Table S2.** Baseline and changes from baseline to the last visit for lipoproteins, UACR and estimated glomerular filtration rate. **Table S3.** Major study end-points: event rates (per 100py) and event rate comparison. **Table S4.** eGFR changes between the baseline and randomization visit. **Figure S1.** Changes from baseline by treatment arm (within each eGFR stratum) for total, LDL and HDL cholesterol, body mass index, creatinine, eGFR, UACR, systolic and diastolic blood pressure, and c-reactive protein.

## Data Availability

The data and materials may be available upon reasonable request.
