# Supplementary material for: Alogliptin after acute coronary syndrome in patients with type 2 diabetes: a renal function stratified analysis of the EXAMINE trial
Source: BMC Med. 2020 Jun 4;18:165. doi: 10.1186/s12916-020-01616-8 (PMC7271537; doi:10.1186/s12916-020-01616-8)
Supplement: Supplementary file 1 — Additional file 1: Table S1. Baseline characteristics of the study population by screening renal function strata. Table S2. Baseline and changes from baseline to the last visit for lipoproteins, UACR and estimated glomerular filtration rate. Table S3. Major study end-points: event rates (per 100py) and event rate comparison. Table S4. eGFR changes between the baseline and randomization visit. Figure S1. Changes from baseline by treatment arm (within each eGFR stratum) for total, LDL and HDL cholesterol, body mass index, creatinine, eGFR, UACR, systolic and diastolic blood pressure, and c-reactive protein. [file 12916_2020_1616_MOESM1_ESM.docx]

**Supplemental Material**

Additional file: Table S1. Baseline characteristics of the study population by screening renal function strata

| **Characteristics** | **eGFR≥60 stratum** | **eGFR<60 stratum** | **p-value** |
| --- | --- | --- | --- |
| N. | 3946 | 1434 |  |
| Age (yrs), mean ± SD | 59.0 ± 9.4 | 66.0 ± 9.5 | <0.001 |
| Age ≥65 yrs | 1103 (28.0%) | 804 (56.1%) | <0.001 |
| Male sex | 2840 (72.0%) | 811 (56.6%) | <0.001 |
| Diabetes duration (yrs), median (IQR) | 6.3 (2.3, 11.8) | 10.3 (4.4, 17.0) | <0.001 |
| BMI (kg/m^2^), mean ± SD | 29.6 ± 5.5 | 29.3 ± 5.8 | 0.080 |
| Race |  |  | <0.001 |
| White | 2935 (74.4%) | 974 (67.9%) |  |
| Asian | 729 (18.5%) | 360 (25.1%) |  |
| Black | 162 (4.1%) | 54 (3.8%) |  |
| Other | 120 (3.0%) | 46 (3.2%) |  |
| Geographic Region |  |  | <0.001 |
| United States, Canada | 626 (15.9%) | 227 (15.8%) |  |
| Mexico, Central/South America | 1014 (25.7%) | 379 (26.4%) |  |
| Western Europe, Australia, New Zealand, Middle East | 474 (12.0%) | 142 (9.9%) |  |
| Eastern Europe, Africa | 1162 (29.4%) | 346 (24.1%) |  |
| Asia/Pacific | 670 (17.0%) | 340 (23.7%) |  |
| Smoking | 627 (15.9%) | 107 (7.5%) | <0.001 |
| Hypertension | 3176 (80.5%) | 1293 (90.2%) | <0.001 |
| Previous MI | 3459 (87.7%) | 1275 (88.9%) | 0.21 |
| PCI | 2501 (63.4%) | 871 (60.7%) | 0.077 |
| CABG | 458 (11.6%) | 230 (16.0%) | <0.001 |
| HF history | 975 (24.7%) | 558 (38.9%) | <0.001 |
| Previous stroke | 234 (5.9%) | 154 (10.7%) | <0.001 |
| PAD | 309 (7.8%) | 205 (14.3%) | <0.001 |
| AFib | 209 (5.3%) | 167 (11.6%) | <0.001 |
| eGFR* (ml/min/1.73m^2^), mean ± SD | 79.5 ± 16.8 | 47.4 ± 13.7 | <0.001 |
| Index ACS Event Type |  |  | 0.36 |
| Myocardial Infarction | 3034 (77.1%) | 1118 (78.2%) |  |
| Unstable Angina | 903 (22.9%) | 311 (21.8%) |  |
| Time from index ACS to randomization, median (IQR) | 44.0 (30.0, 63.0) | 43.0 (30.0, 65.0) | 0.90 |
| Troponin I (ng/L), median (IQR) | 7.7 (4.0, 16.9) | 13.3 (6.7, 29.2) | <0.001 |
| Heart rate (bpm), mean ± SD | 71.5 ± 10.6 | 70.8 ± 11.2 | 0.031 |
| SBP (mmHg), mean ± SD | 128.2 ± 16.3 | 131.1 ± 17.4 | <0.001 |
| DBP (mmHg), mean ± SD | 76.8 ± 9.4 | 75.4 ± 10.2 | <0.001 |
| Total cholesterol (mg/dL), mean ± SD | 152.6 ± 42.7 | 159.2 ± 46.5 | <0.001 |
| LDL cholesterol (mg/dL), mean ± SD | 43.0 ± 10.4 | 43.4 ± 11.2 | 0.23 |
| HDL cholesterol (mg/dL), mean ± SD | 77.3 ± 33.4 | 82.2 ± 37.9 | <0.001 |
| Triglycerides (mg/dL), mean ± SD | 163.2 ± 107.1 | 168.2 ± 94.5 | 0.12 |
| UACR (mg/g creat.), median (IQR) | 3.4 (1.6, 10.0) | 9.7 (2.9-50.6) | <0.001 |
| C-reactive protein (mg/dL), mean ± SD | 5.1 ± 12.3 | 6.9 ± 17.0 | <0.001 |
| Antiplatelet agents | 3844 (97.4%) | 1388 (96.8%) | 0.22 |
| Beta-blockers | 3247 (82.3%) | 1164 (81.2%) | 0.35 |
| ACEi/ARBs | 3267 (82.8%) | 1144 (79.8%) | 0.011 |
| Statins | 3594 (91.1%) | 1272 (88.7%) | 0.009 |
| Antidiabetic agents | 3916 (99.2%) | 1409 (98.3%) | 0.002 |
| Insulin | 1076 (27.3%) | 529 (36.9%) | <0.001 |
| Metformin | 2861 (72.5%) | 701 (48.9%) | <0.001 |
| Thiazolidinediones | 83 (2.1%) | 48 (3.3%) | 0.009 |
| Sulfonylureas | 1873 (47.5%) | 630 (43.9%) | 0.022 |
| Calcium channel blockers | 763 (19.3%) | 434 (30.3%) | <0.001 |
| Diuretics (any) | 1240 (31.4%) | 774 (54.0%) | <0.001 |

Legend: MI, myocardial infarction; PCI, percutaneous coronary intervention; CABG, coronary-artery bypass grafting; HF, heart failure; PAD, peripheral artery disease; AFib, atrial fibrillation; eGFR, estimated glomerular filtration rate; ACS, acute coronary syndrome; SBP, systolic blood pressure; DBP diastolic blood pressure; ACEi/ARBs, angiotensin converting enzyme inhibitors/angiotensin receptor blockers; UACR, urinary albumin-to-creatinine ratio.

*eGFR at randomization, that occurred 9 (7-13) days after the baseline visit.

Stratification according to renal function was performed at the baseline visit, as follows: 1) “normal renal function” stratum if eGFR ≥60 ml/min/1.73m^2^ or 2) “impaired renal function” stratum if eGFR <60 ml/min/1.73m^2^.

Additional file: Table S2. Baseline and changes from baseline to the last visit for lipoproteins, UACR and estimated glomerular filtration rate

| **Variable change** | **Placebo** | **Alogliptin** | **P-value*** | **Inter. P** |
| --- | --- | --- | --- | --- |
| ***eGFR ≥60 stratum*** | | | | |
| Total cholesterol (mg/dL) |  |  |  |  |
| Baseline | 153±42 | 152±43 | 0.19 |  |
| Last visit: change from baseline | 14±44 | 14±44 | 0.62 | 0.56 |
| HDL cholesterol (mg/dL) |  |  |  |  |
| Baseline | 43±10 | 43±11 | 0.25 |  |
| Last visit: change from baseline | 1.7±8.4 | 1.5±8.6 | 0.34 | 0.23 |
| LDL cholesterol (mg/dL) |  |  |  |  |
| Baseline | 78±34 | 77±33 | 0.17 |  |
| Last visit: change from baseline | 9.3±34.4 | 9.0±33.6 | 0.78 | 0.27 |
| Triglycerides (mg/dL) |  |  |  |  |
| Baseline | 165±108 | 161±105 | 0.18 |  |
| Last visit: change from baseline | 21±171 | 17±142 | 0.48 | 0.49 |
| eGFR change from baseline | 0.3±15 | -0.4±14 | 0.11 | 0.15 |
| U albumin/creatinine ratio (mg/g of creatinine) |  |  |  |  |
| Baseline | 17.5±54.1 | 18.1±69.1 | 0.83 |  |
| 12 months: change from baseline | 1.7±7.1 | 1.0±4.1 | 0.042 | 0.96 |
| ***eGFR<60 stratum*** | | | | |
| Total cholesterol (mg/dL) |  |  |  |  |
| Baseline | 158±46 | 160±47 | 0.62 |  |
| Last visit: change from baseline | 10±47 | 9±41 | 0.55 | 0.56 |
| HDL cholesterol (mg/dL) |  |  |  |  |
| Baseline | 44±11 | 43±11 | 0.25 |  |
| Last visit: change from baseline | 1.7±9.7 | 1.7±9.7 | 0.94 | 0.23 |
| LDL cholesterol (mg/dL) |  |  |  |  |
| Baseline | 81±37 | 83±39 | 0.24 |  |
| Last visit: change from baseline | 6.8±38.8 | 4.7±33.4 | 0.29 | 0.27 |
| Triglycerides (mg/dL) |  |  |  |  |
| Baseline | 168±98 | 167±91 | 0.84 |  |
| Last visit: change from baseline | 7±93 | 11±99 | 0.39 | 0.49 |
| eGFR change from baseline | 0.1±11 | -0.9±11 | 0.11 | 0.15 |
| U albumin/creatinine ratio (mg/mmol of creatinine) |  |  |  |  |
| Baseline | 67.8±152 | 74.1±167 | 0.53 |  |
| 12 months: change from baseline | 2.4±17.3 | 5.1±46.4 | 0.33 | 0.96 |

Legend: HDL, high-density lipoprotein; LDL, low-density lipoprotein; eGFR, estimated glomerular filtration rate in ml/min/1.73m^2^; Inter. P, p-value for interaction between eGFR strata and treatment allocation for each outcome in a linear regression model.

*non-parametric comparisons provide similar results.

Additional file: Table S3. Major study end-points: event rates (per 100py) and event rate comparison

| End-point | Placebo | Alogliptin | ARD (95%CI) | P-value* |
| --- | --- | --- | --- | --- |
| ***eGFR ≥60 stratum*** | | | | |
| Primary end-point | 6.5 (5.6-7.4) | 5.2 (4.4-6.1) | -1.3 (-2.4 to -0.4) | 0.042 |
| Components of the primary end-point | | | | |
| Cardiovascular death | 1.9 (1.5-2.5) | 1.2 (0.8-1.62) | -0.7 (-1.4 to -0.1) | 0.018 |
| Non-fatal MI | 3.8 (3.1-4.5) | 3.3 (2.7-4.0) | -0.5 (-1.4 to +0.4) | 0.27 |
| Non-fatal stroke | 0.6 (0.4-1.0) | 0.7 (0.4-1.0) | +0.1 (-0.3 to +0.5) | 0.78 |
| Secondary end-point | 7.7 (6.8-8.8) | 6.3 (5.5-7.3) | -1.4 (-2.8 to -0.1) | 0.042 |
| All-cause death | 2.9 (2.3-3.5) | 2.3 (1.9-2.9) | -0.5 (-1.3 to +0.3) | 0.22 |
| All cardiovascular deaths | 2.3 (1.8-2.9) | 1.4 (1.0-1.9) | -0.9 (-1.6 to -0.2) | 0.008 |
| Heart failure hospitalization | 1.4 (1.0-1.9) | 1.4 (1.1-1.9) | -0.01 (-0.5 to +0.6) | 0.95 |
| ***eGFR <60 stratum*** | | | | |
| Primary end-point | 12.1 (10.2-14.5) | 14.6 (12.5-17.2) | +2.5 (-0.7 to +5.7) | 0.13 |
| Components of the primary end-point | | | | |
| Cardiovascular death | 4.7 (3.5-6.1) | 4.7 (3.6-6.2) | +0.1 (-1.8 to +1.9) | 0.95 |
| Non-fatal MI | 5.8 (4.5-7.5) | 8.6 (7.0-10.6) | +2.8 (+0.5 to +5.1) | 0.017 |
| Non-fatal stroke | 1.2 (0.7-2.1) | 0.7 (0.4-1.5) | -0.5 (-1.3 to +0.4) | 0.28 |
| Secondary end-point | 13.2 (11.1-15.6) | 15.5 (13.2-18.1) | +2.3 (-0.9 to + 5.6) | 0.17 |
| All-cause death | 7.7 (6.2-9.5) | 7.2 (5.7-8.9) | -0.5 (-2.7 to +1.8) | 0.66 |
| All cardiovascular deaths | 5.3 (4.1-6.8) | 6.2 (4.9-7.8) | +0.9 (-1.1 to +2.3) | 0.40 |
| Heart failure hospitalization | 4.4 (3.3-5.8) | 5.9 (4.6-7.6) | +1.6 (-0.3 to +3.5) | 0.12 |

*P-values for interaction, as follows:

- Primary outcome: treatment allocation (alogliptin or placebo) * eGFR stratum, interaction P =0.018

- Cardiovascular death: treatment allocation (alogliptin or placebo) * eGFR stratum, interaction P =0.076

- Non-fatal MI: treatment allocation (alogliptin or placebo) * eGFR stratum, interaction P =0.016

- Non-fatal stroke: treatment allocation (alogliptin or placebo) * eGFR stratum, interaction P =0.29

- Secondary end-point: treatment allocation (alogliptin or placebo) * eGFR stratum, interaction P =0.028

- All-cause death: treatment allocation (alogliptin or placebo) * eGFR stratum, interaction P =0.57

- All cardiovascular deaths: treatment allocation (alogliptin or placebo) * eGFR stratum, interaction P =0.012

- Heart failure hospitalization: treatment allocation (alogliptin or placebo) * eGFR stratum, interaction P =0.33

The primary end-point was a composite of cardiovascular death, non-fatal MI and non-fatal stroke

The secondary end point was a composite of death from cardiovascular causes, nonfatal myocardial infarction, nonfatal

stroke, or urgent revascularization due to unstable angina within 24 hours after hospital admission.

Legend: MI, myocardial infarction; ARD, absolute risk difference.

Additional file: Table S4. eGFR changes between the baseline and randomization visit

| **Placebo group** | | |
| --- | --- | --- |
| eGFR categories | Baseline: eGFR ≥60 | Baseline: eGFR <60 |
| Randomization: eGFR ≥60 | 1769 (90.1%) | 117 (16.3%) |
| Randomization: eGFR <60 | 194 (9.9%) | 599 (83.4%) |
| **Alogliptin group** | | |
| eGFR categories | Baseline: eGFR ≥60 | Baseline: eGFR <60 |
| Randomization: eGFR ≥60 | 1812 (91.4%) | 117 (16.3%) |
| Randomization: eGFR <60 | 171 (8.6%) | 601 (83.7%) |

eGFR, estimated glomerular filtration rate in ml/min/1.73m2

eGFR at randomization occurred 9 (7-13) days after the baseline visit where the stratification was performed.

Additional file: Figure S1. Changes from baseline by treatment arm (within each eGFR stratum) for total, LDL and HDL cholesterol, body mass index, creatinine, eGFR, UACR, systolic and diastolic blood pressure, and c-reactive protein.

1. Total cholesterol


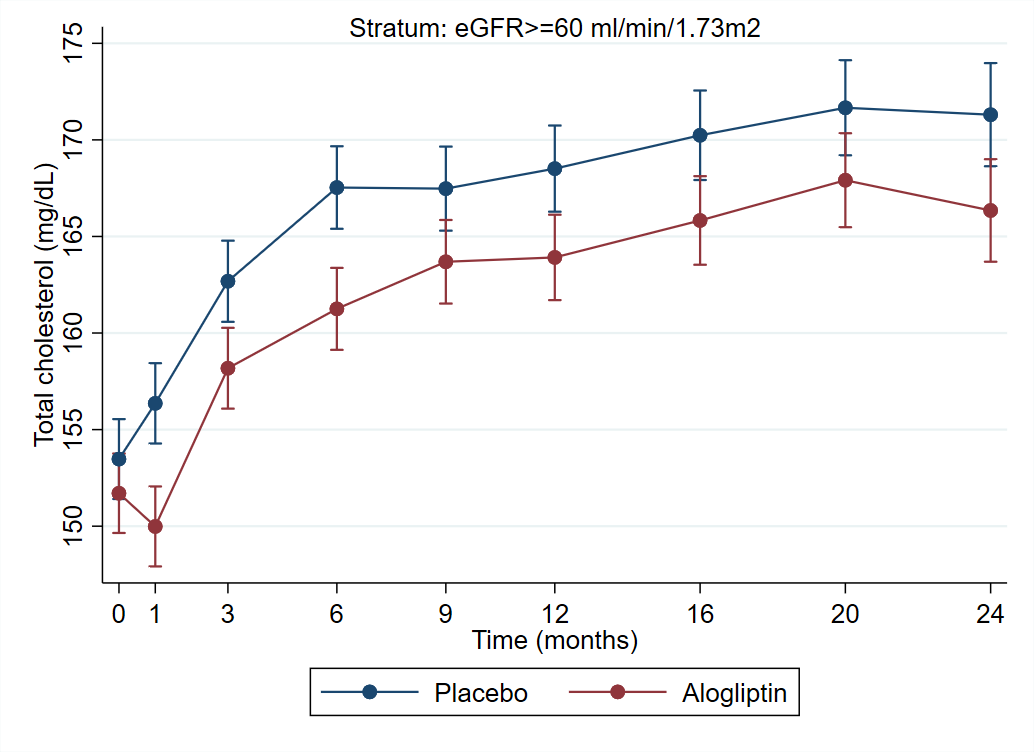


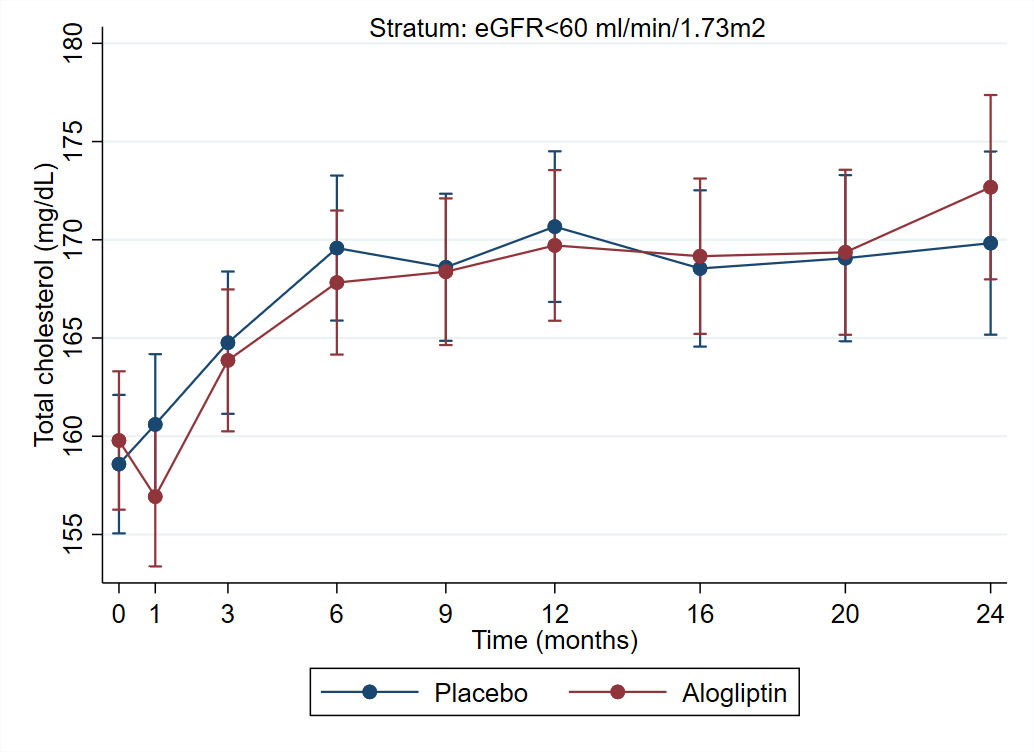


1. HDL cholesterol


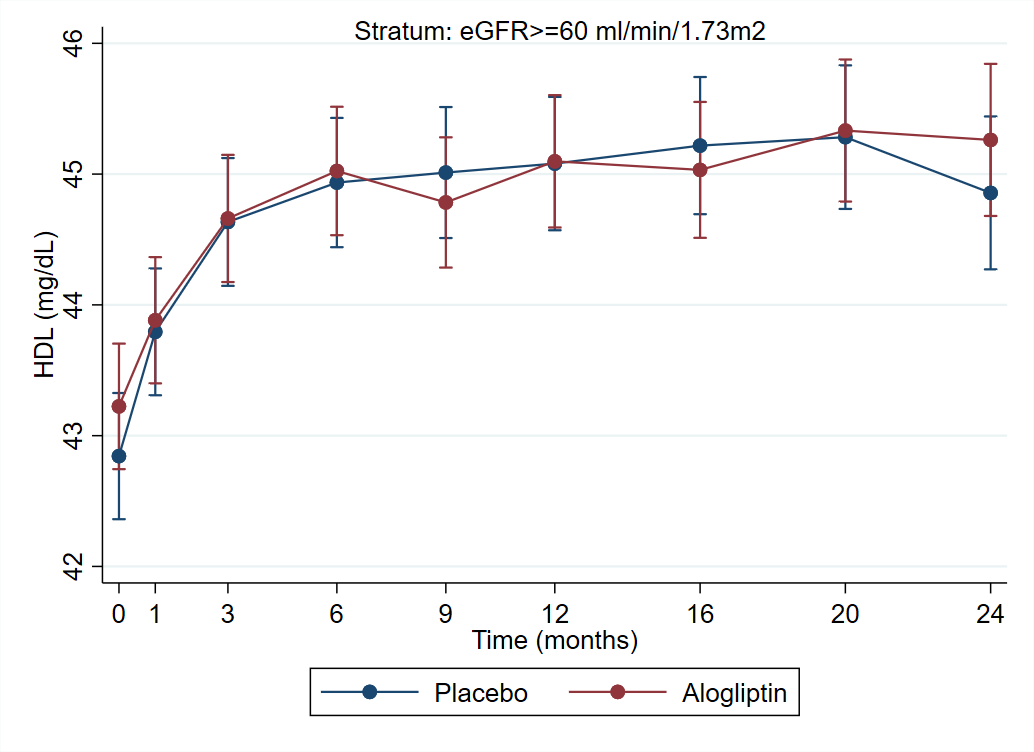


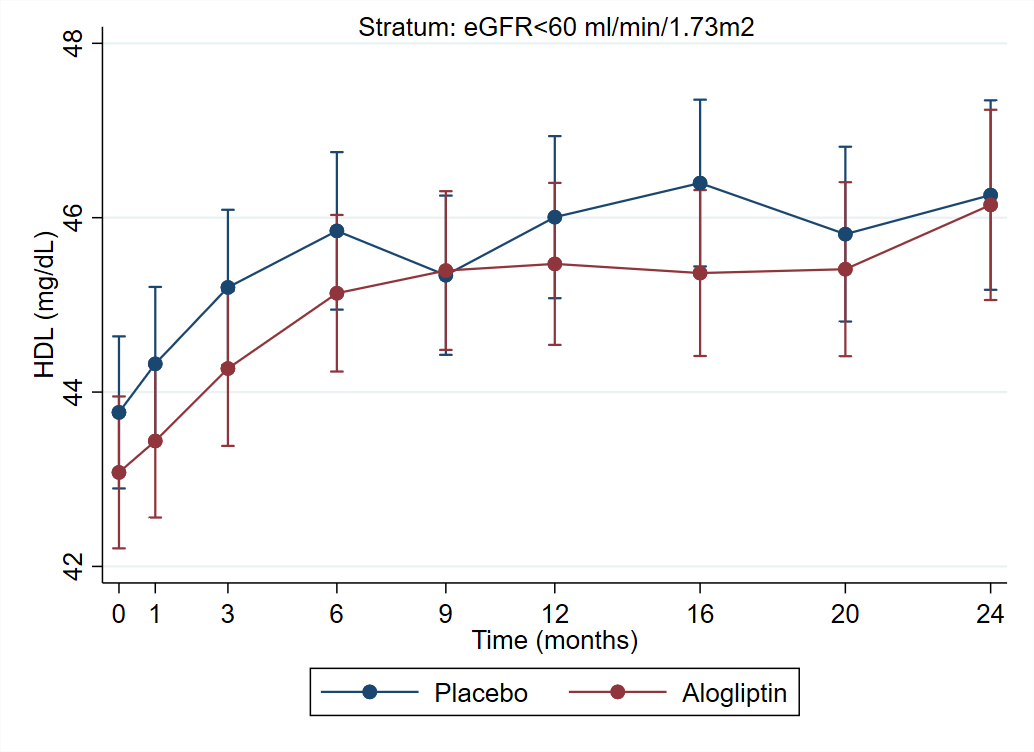


1. LDL cholesterol


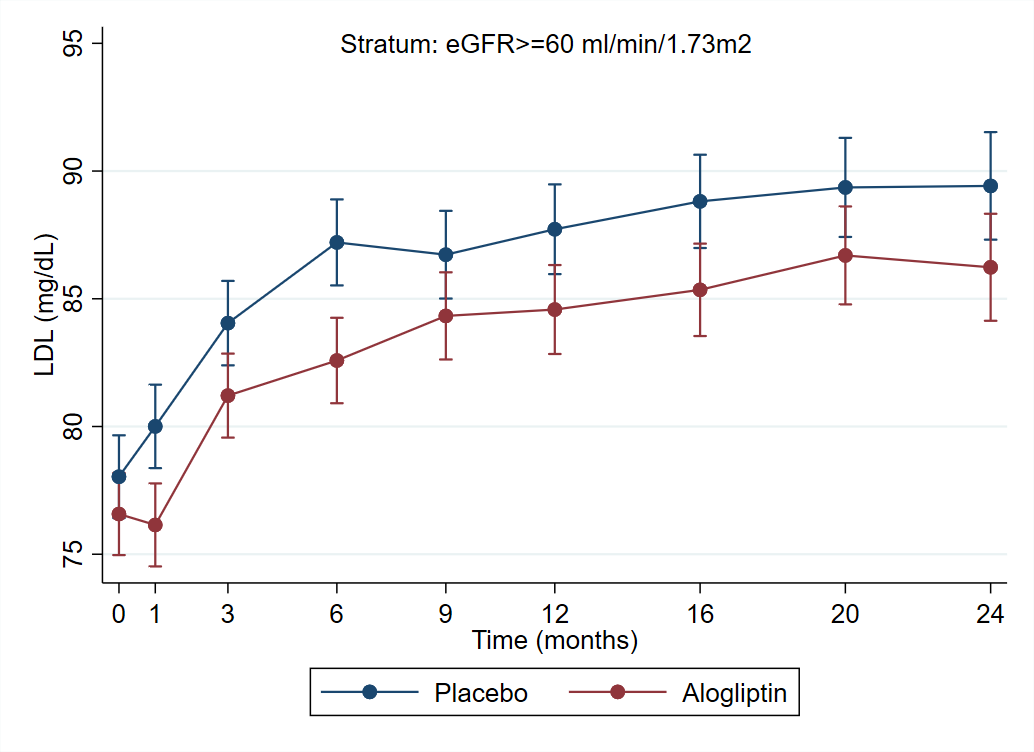


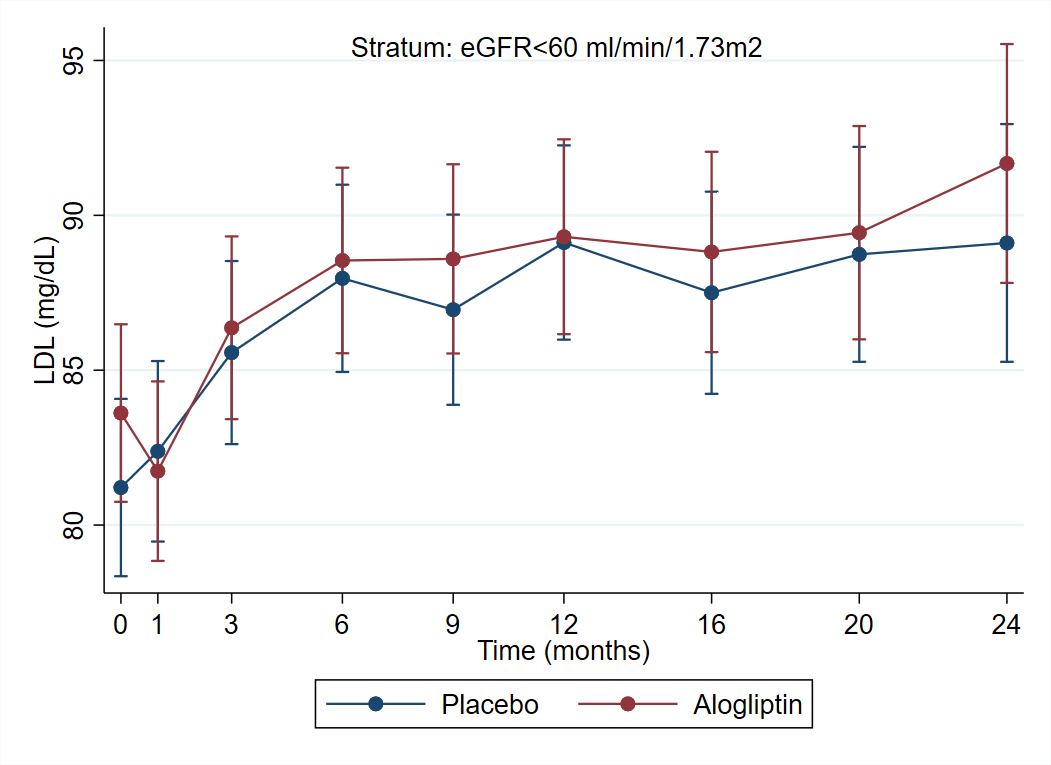


1. Triglycerides


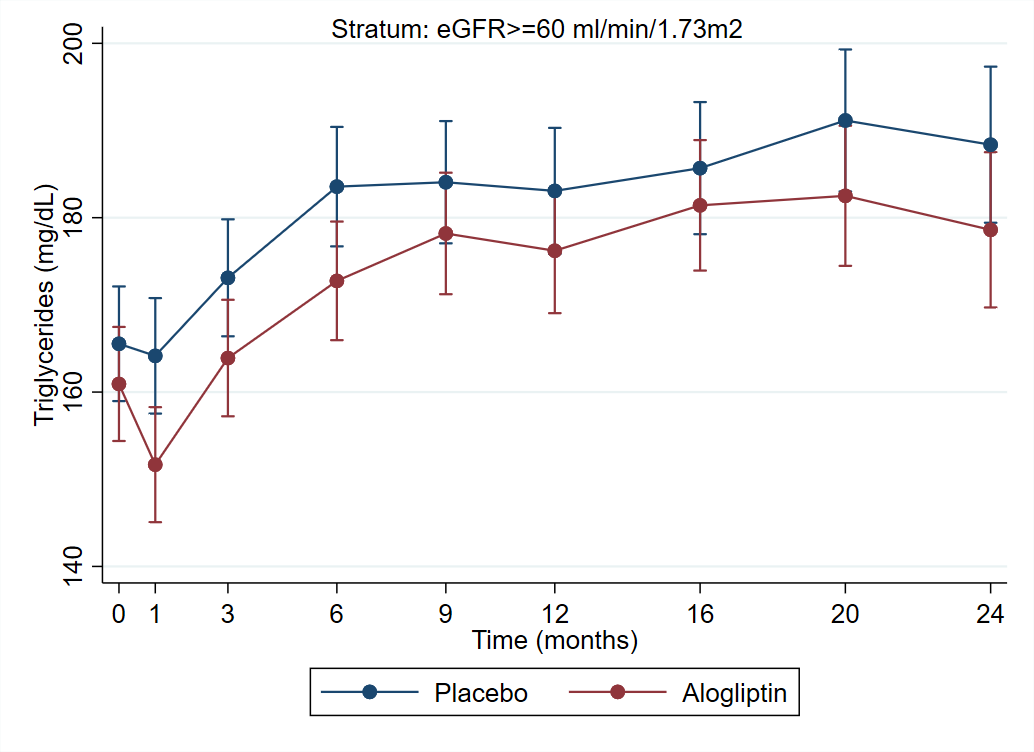


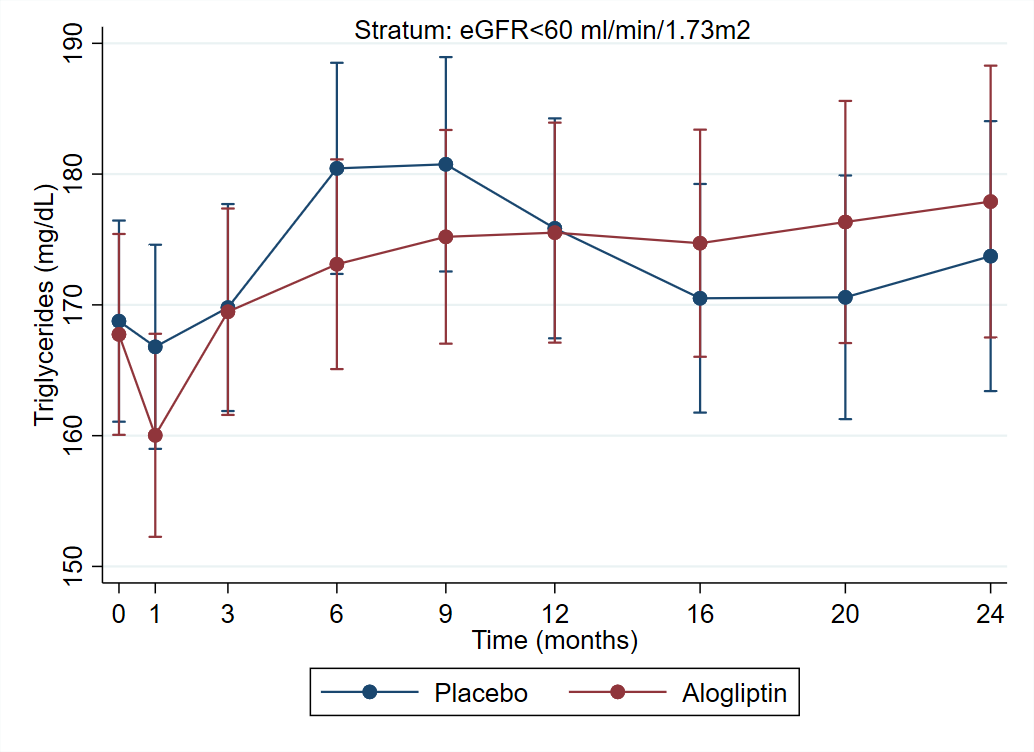


1. SBP


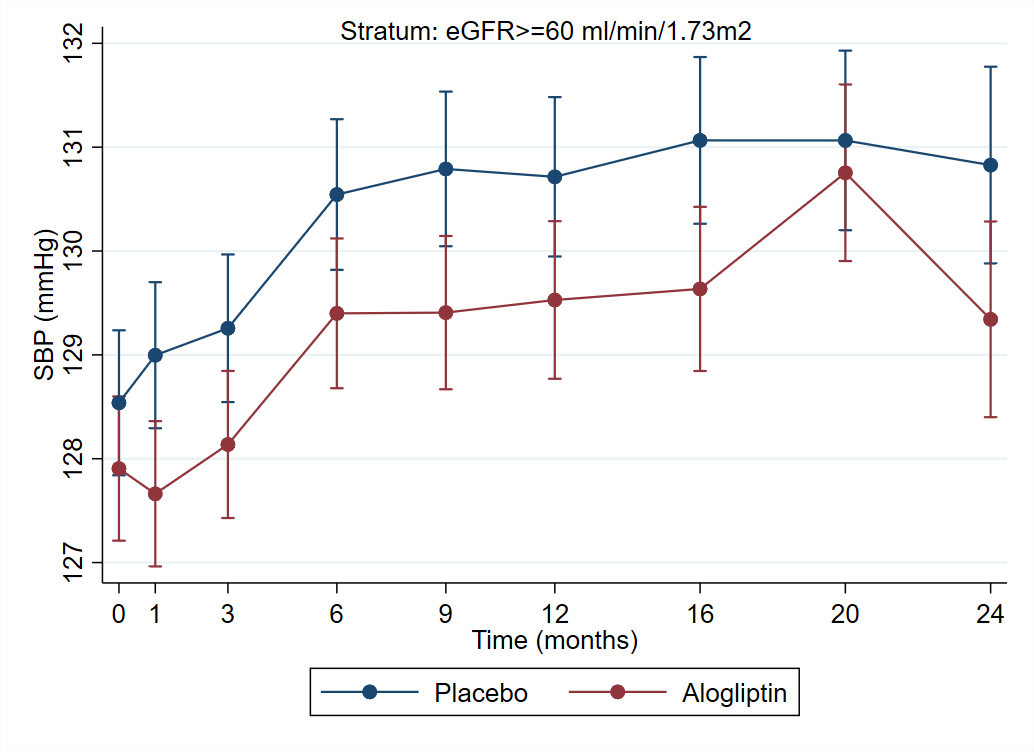

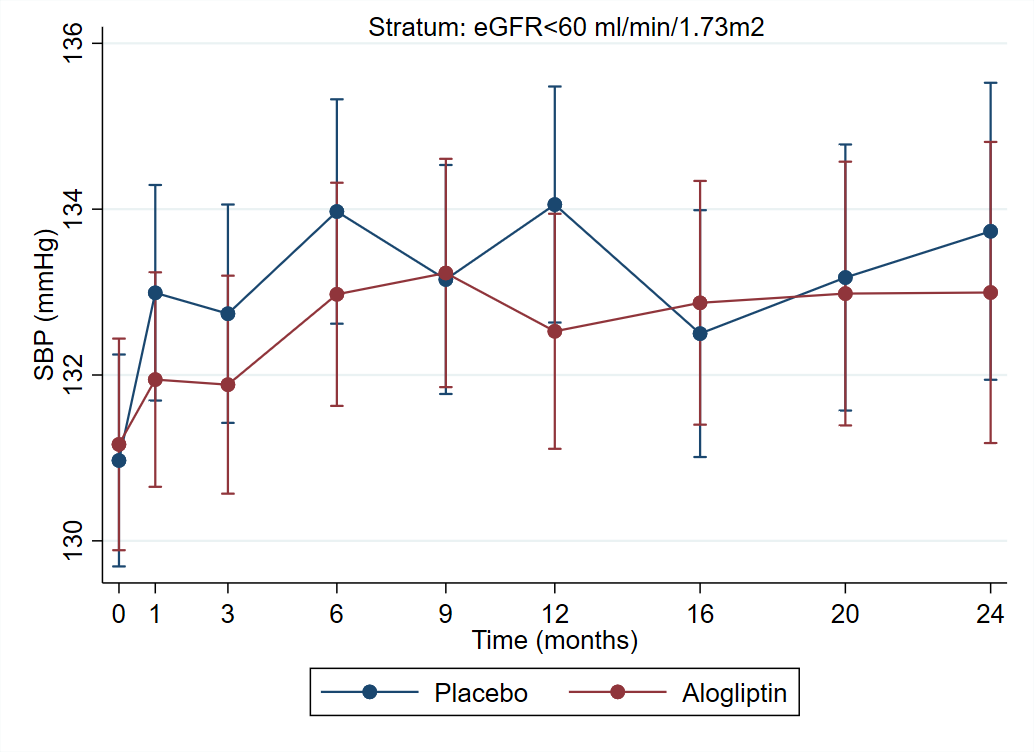


1. DBP


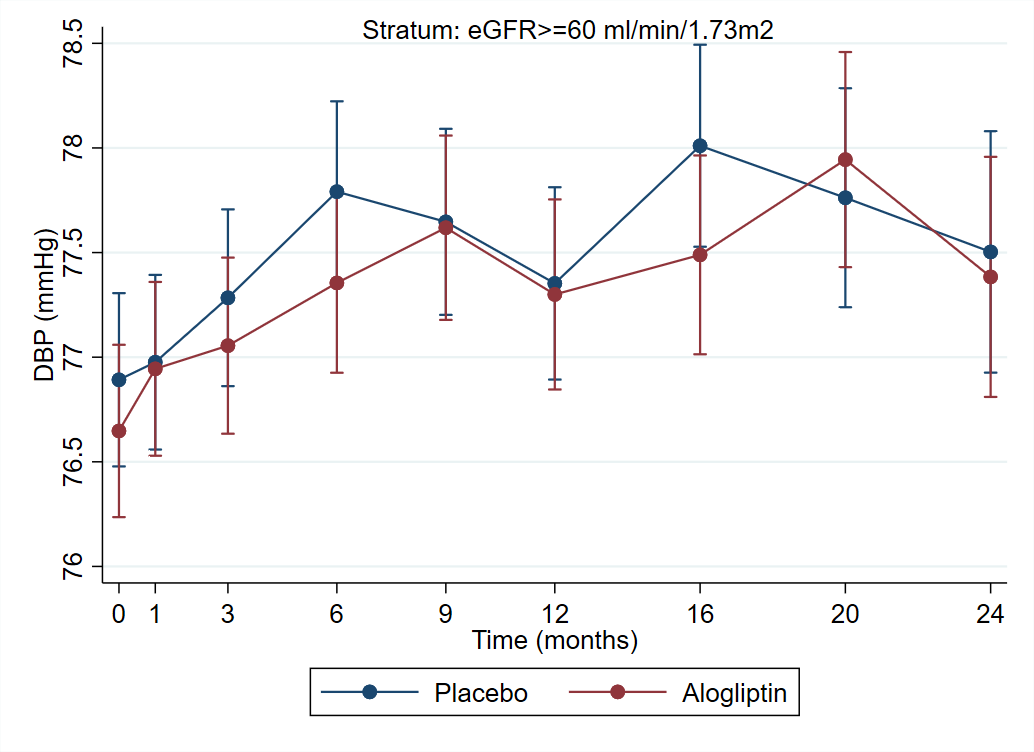

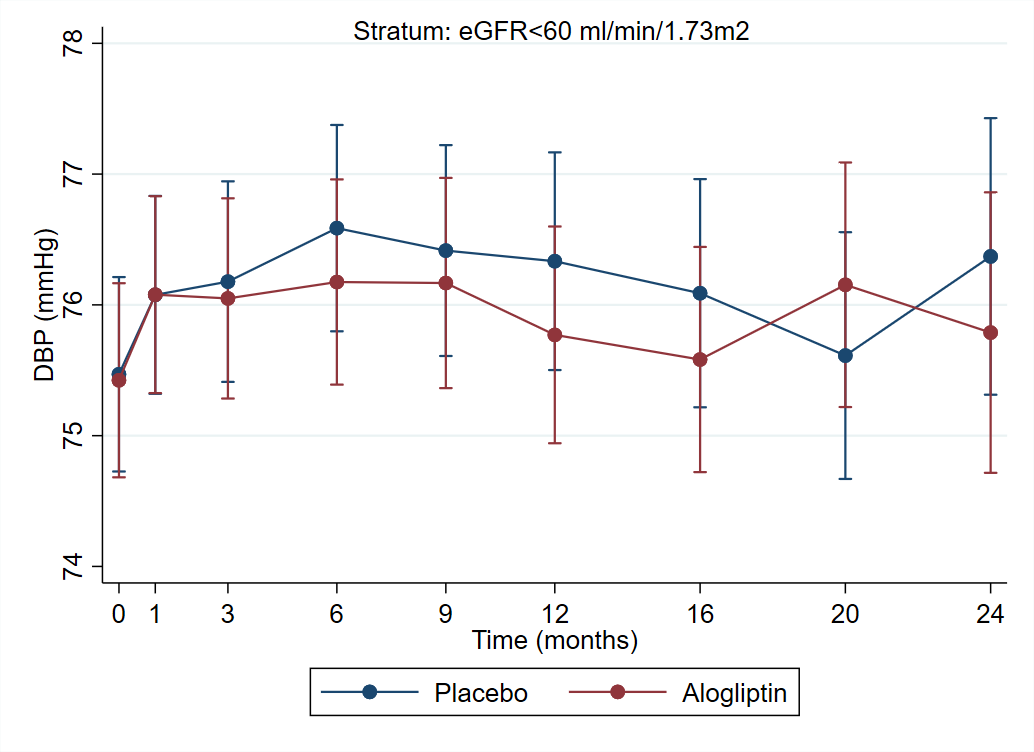


1. BMI


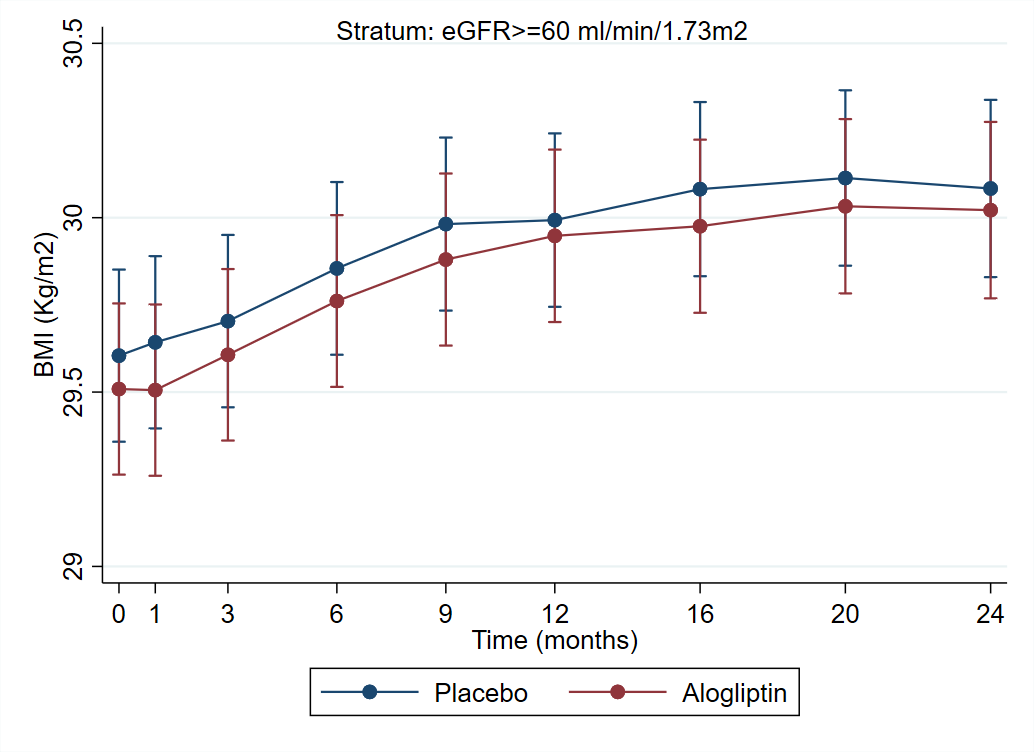

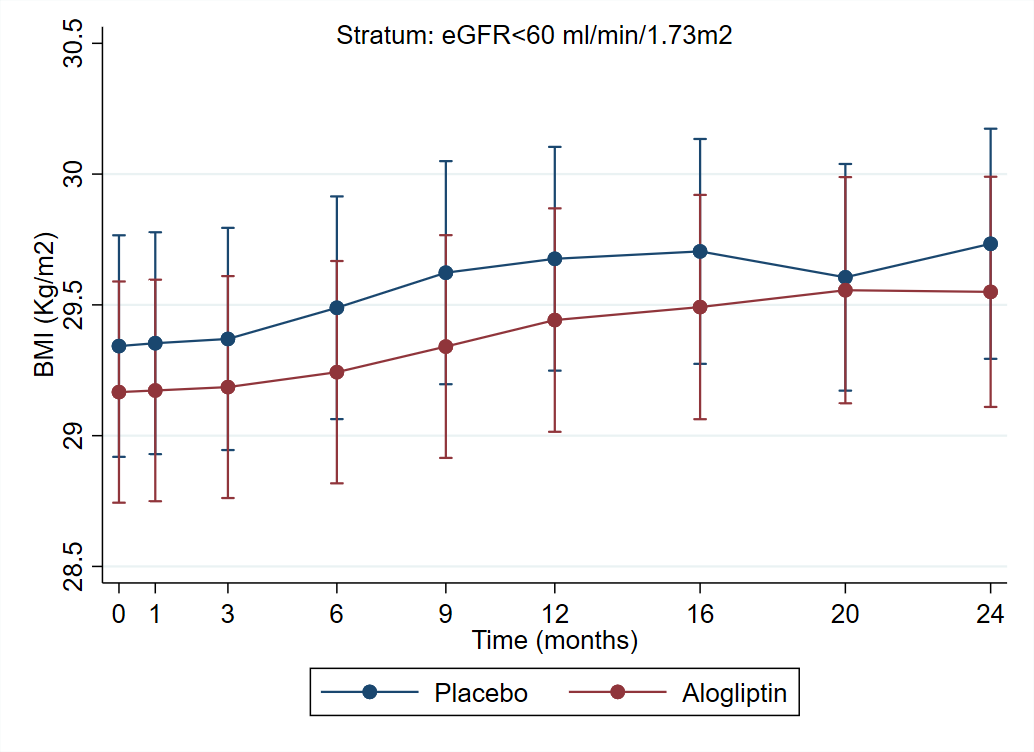


1. Creatinine


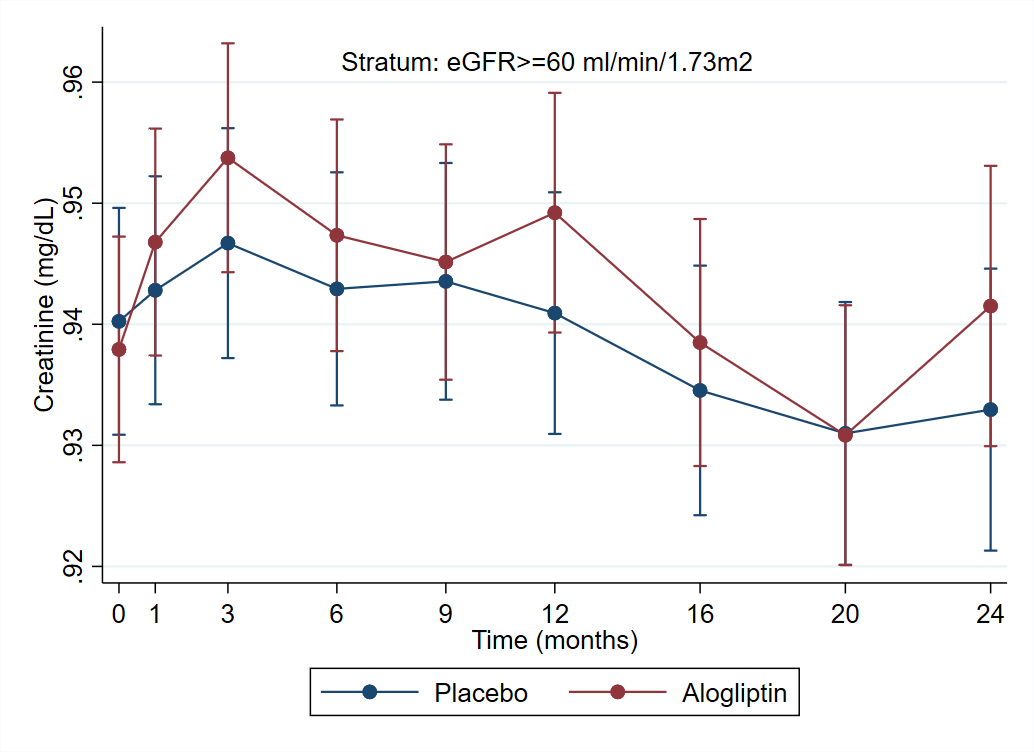

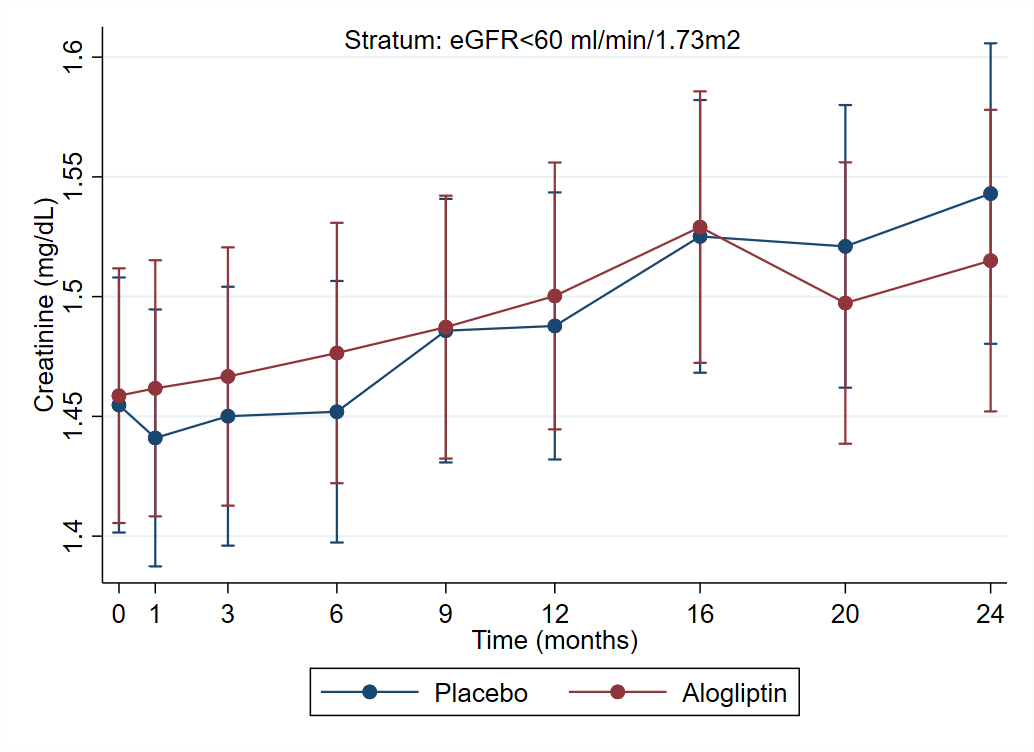


1. eGFR


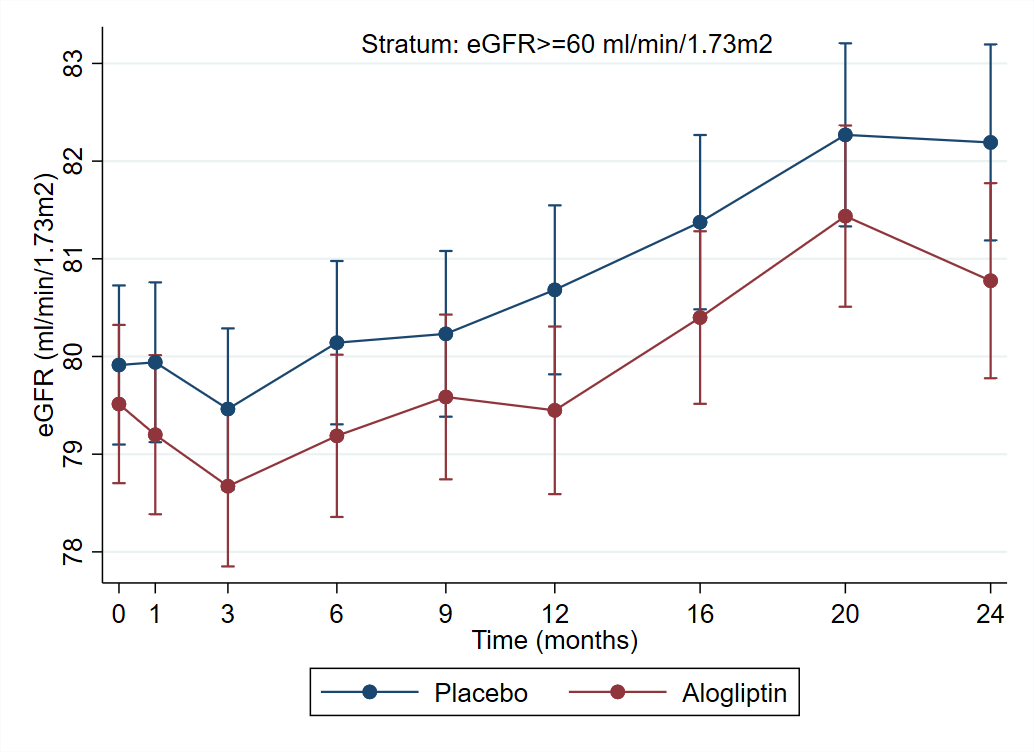

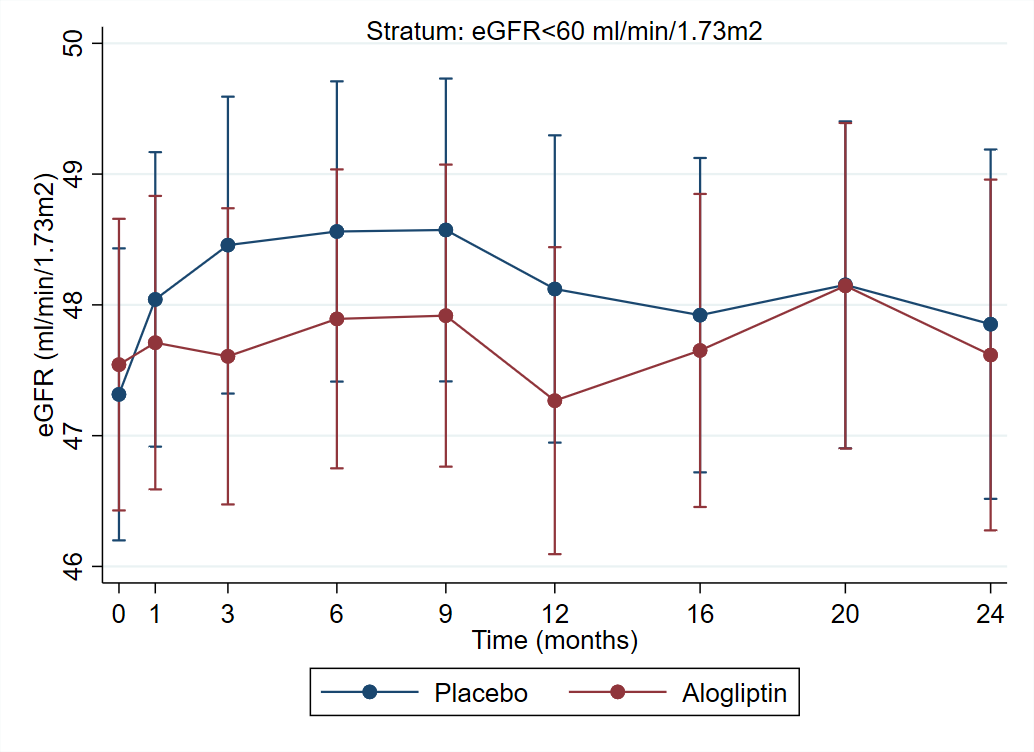


1. UACR


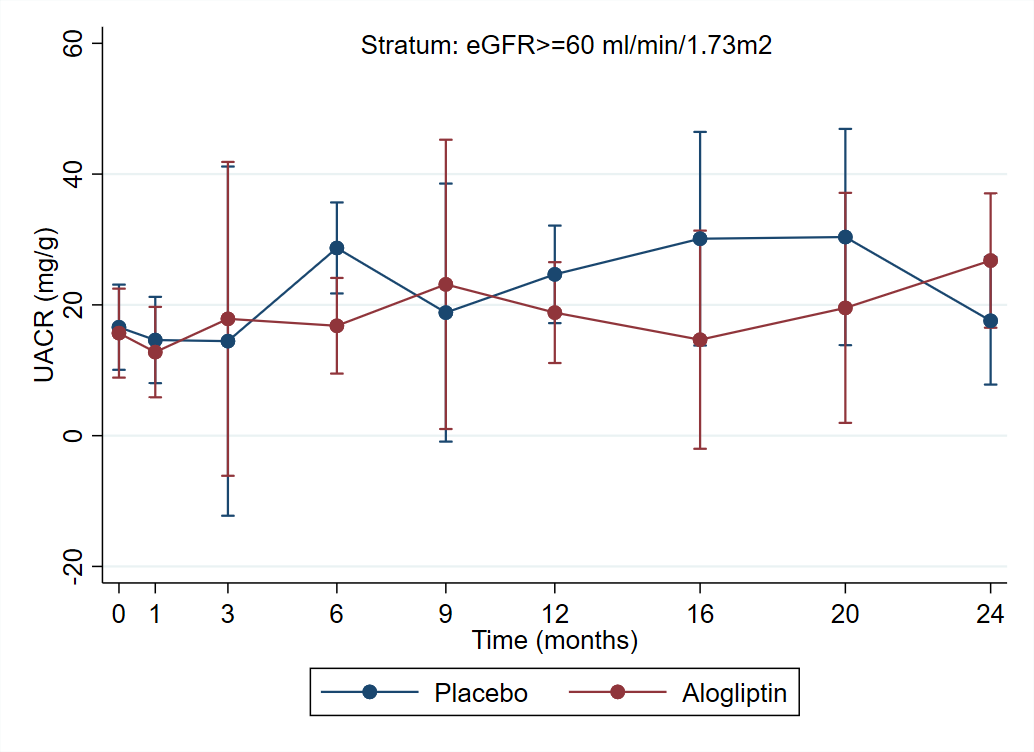

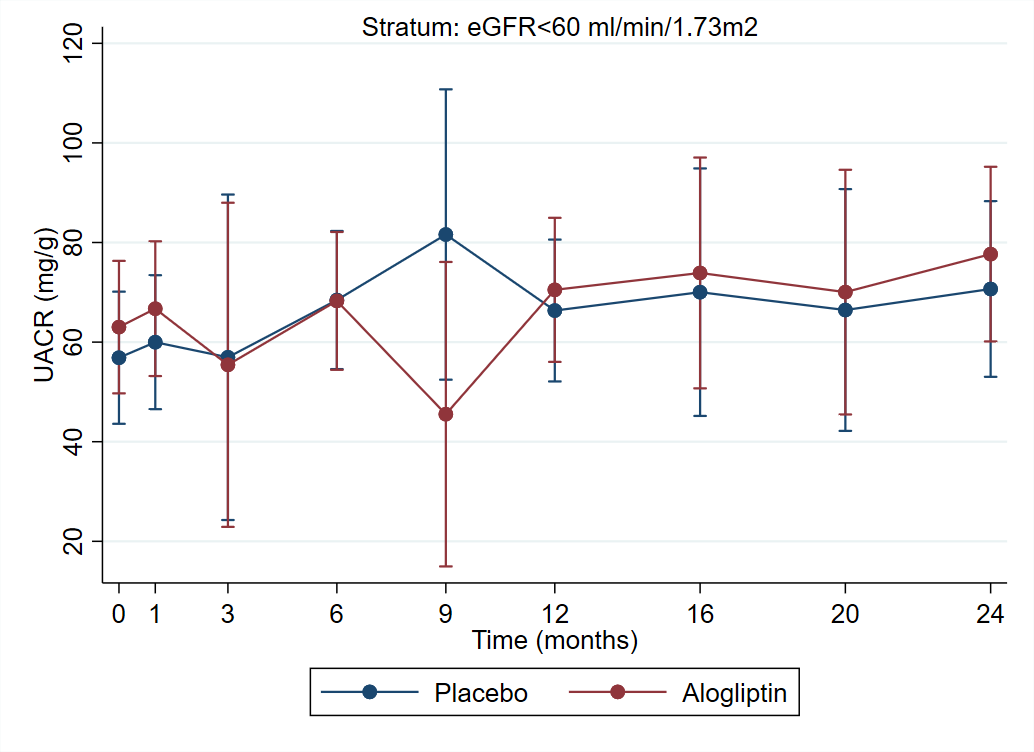


1. C-reactive protein


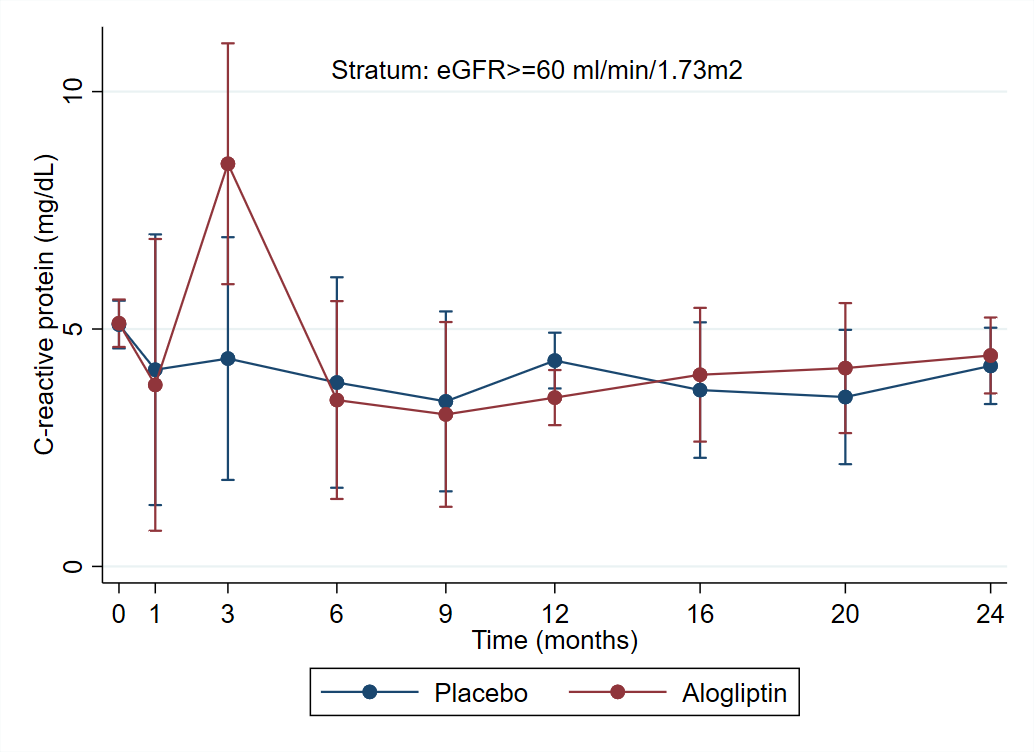

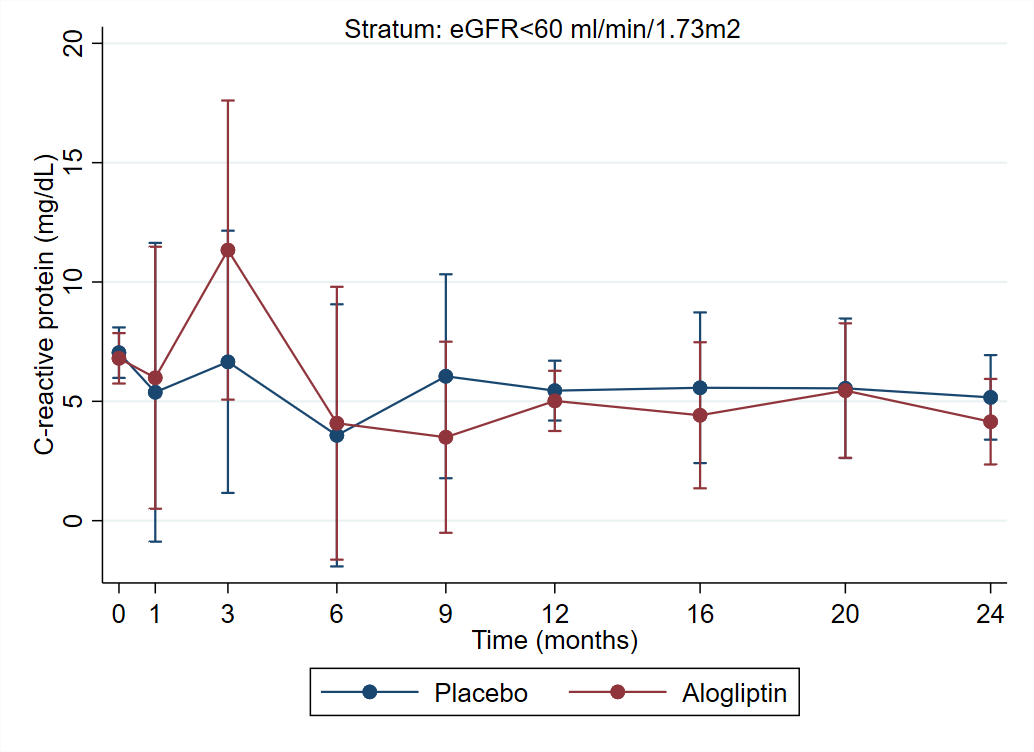


P-value >0.05 for all comparisons (multiple comparison adjusted global effect of alogliptin vs. placebo).

The p-values for each measure within the eGFR strata are:

eGFR≥60:

- Total cholesterol =0.23
- HDL cholesterol =0.39
- LDL cholesterol =0.14
- Triglycerides =0.73
- SBP =0.58
- DBP =0.70
- BMI =0.86
- Creatinine =0.62
- eGFR =0.76
- UACR =0.34
- C-reactive protein =0.29

eGFR<60:

- Total cholesterol =0.42
- HDL cholesterol =0.52
- LDL cholesterol =0.84
- Triglycerides =0.24
- SBP =0.61
- DBP =0.86
- BMI =0.67
- Creatinine =0.75
- eGFR =0.54
- UACR =0.76
- C-reactive protein =0.96

Legend: SBP, systolic blood pressure; DBP, diastolic blood pressure; BMI, body mass index; eGFR, estimated glomerular filtration rate; UACR, urinary albumin-to-creatinine ratio.
